# Dynamic Polarization Control of Nonlinear Terahertz Photoresponse via Topological Phase Transitions

**DOI:** 10.34133/research.0899

**Published:** 2025-09-25

**Authors:** Libo Zhang, Xuyang Lv, Zhuo Dong, Debasis Dutta, Liu Yang, Raihan Ahammed, Atasi Chakraborty, Dong Wang, Zhen Hu, Mengjie Jiang, Kaixuan Zhang, Li Han, Kai Zhang, Amit Agarwal, Xiaoshuang Chen, Lin Wang

**Affiliations:** ^1^ College of Physics and Optoelectronic Engineering, Hangzhou Institute for Advanced Study, University of Chinese Academy of Sciences, Hangzhou 310024, China.; ^2^ State Key Laboratory of Infrared Physics, Shanghai Institute of Technical Physics, Chinese Academy of Sciences, Shanghai 200083, China.; ^3^ Key Laboratory of Semiconductor DisplayMaterials and Chips & *i*-Lab, Suzhou Institute of Nano-Tech and Nano-Bionics (SINANO), Chinese Academy of Sciences, Suzhou 215123, China.; ^4^ Department of Physics, Indian Institute of Technology Kanpur, Kanpur 208016, India.; ^5^ Institute für Physik, Johannes-Gutenberg-Universität Mainz, D-55099 Mainz, Germany.; ^6^ College of Optical and Electronic Technology, China Jiliang University, Hangzhou 310018, China.; ^7^ Suzhou Laboratory, Suzhou, Jiangsu 215000, China.

## Abstract

Precise modulation of topologically protected states via external stimuli, such as electric, optical, and magnetic fields, is a cornerstone for advancing robust topological photonics and quantum technologies. However, the realization of dynamic and noninvasive control remains constrained by the high-energy thresholds of conventional stimuli, which can disrupt delicate topological states. Here, we employ low-energy terahertz excitation to directly probe the photoresponse across a temperature-induced topological phase transition in ultrathin ZrTe_5_, a material at the intersection of topological physics and low-dimensional systems, leveraging its unique ability to interact with low-energy quasiparticle states without compromising coherence in the system. We observe a giant and robust nonlinear terahertz photoresponse characterized by in situ tunable geometric properties of Bloch quasiparticles. The response exhibits colossal behavior and a sign reversal across a temperature-driven topological phase transition, linked to a nonvanishing Berry curvature dipole that serves as a direct marker of symmetry-breaking evolution between weak (*m* < 0) and strong (*m* > 0) topological insulator phases. The observed device exhibits a response time of ~1 μs with a noise equivalent power of 5.6 pW/Hz^0.5^ across the 0.5-THz range, demonstrating the potential of topological phase transitions for terahertz detection. These findings underscore the potential of low-energy terahertz excitation for dynamically polarizing and controlling topological states in ultrathin materials, offering a versatile framework for exploring symmetry-breaking phenomena and advancing next-generation optoelectronic devices.

## Introduction

The advent of topological insulator (TI), a new quantum state of matter, that is characterized by an ordinary insulating gap in the bulk while having a metallic spin-polarized surface state (or gapless edge), protected by time-reversal symmetry, has observably marked a watershed moment in condensed matter physics and materials science [1–5]. In the last decades, quite a few categories of topological quantum materials have been explored, including type-I/type-II Dirac and Weyl semimetals, which are characterized by the nontrivial gapless bulk bands and their associated surface states [6–8]. Owing to exotic optoelectronic and optospintronic properties of helical Dirac fermions in gapless topological surface states, polarized photocurrent governed by circularly/linearly polarized high-energy photons exhibits sensitivity dependent on the helicity of the photon and the spin orientation of the surface state [9–11]. Quantum nonlinear responses, including shift current, nonreciprocal charge transport, and optical Hall effect, have been extensively studied to reveal symmetry and the geometric phase of the Bloch wavefunctions in a crystalline solid [12–18]. Several definite features of the Bloch quasiparticle related to quantum geometry effects can be tuned via various parameters in quantum materials [19–23], such as miniband folding via Bragg scattering of electrons, Berry curvature, and associated nonlinear phenomena under dynamic tuning, as well as applications in spintronics, quantum computing, and advanced photonic devices [24–26]. However, there is a notable lack of exploration on the precise tuning of symmetry-broken states and their correlation with nonlinear responses, limiting our understanding and potential exploitation of these phenomena.

The terahertz spectrum (0.1 to 10 THz) holds transformative potential for next-generation technologies, including 6G communication and quantum sensing, owing to the unique alignment with both the vibrational fingerprints of biomolecules for label-free sensing and the ultrafast carrier dynamics essential for programmable devices [27–30]. However, its full exploitation is hindered by several critical bottlenecks in detector technology. First, at a fundamental level, room-temperature detection is challenged by a thermodynamic conflict where the terahertz photon energy (4 to 40 meV) is comparable to the ambient thermal energy (~25 meV). This intrinsic noise floor means that commercial devices, such as thermal bolometers, typically exhibit a poor noise equivalent power (NEP) of ≥1 nW/Hz^1/2^, rendering them inadequate for capturing weak signals in remote sensing or non-line-of-sight communication. Second, bandwidth and speed are restricted. Resonant detectors like quantum cascade devices operate within narrow frequency windows, limiting their utility in broadband spectroscopy, while achieving a high-speed response at higher frequencies is further complicated by complex, nonlinear carrier dynamics that impede device optimization. Third, existing detectors lack dynamic tunability, relying on fixed band structures that prevent the in situ switching of response polarity or magnitude—a critical capability for adaptive 6G transceivers that must adjust to dynamic channel conditions. While cryogenic detectors can overcome some of these performance issues, their reliance on bulky, power-intensive cooling systems makes them impractical for the portable, field-deployed applications driving much of the current demand.

Theoretical studies have highlighted the layered material zirconium pentatelluride (ZrTe_5_) [31–33], which serves as an exemplary material for studying critical phenomena during topological phase transitions (TPTs), spanning from weak topological insulator (WTI), Dirac semimetal (DSM), to strong topological insulator (STI) phases [34]. Considerable efforts have been devoted to exploring the nonequilibrium responses to electromagnetic phenomena, such as spin-polarized electrical currents and the topological magnetoelectric effect, emerging from quantum geometry and topology [35–38]. Harnessing the topological invariants by different external fields (strain [39], magnetism [40], light [23,41,42], etc.) in these materials enables the new insights into Berry physics-related topology in the electronic state, alongside the polarity and amplitude controls observed in photodetectors. Experimental validation through techniques such as scanning tunneling microscopy (STM) [43], angle-resolved photoelectron spectroscopy (ARPES) [30,44], and infrared spectroscopy corroborates these theoretical predictions [45]. Conversely, the TPT, predominantly driven by the inversion of conduction (CB) and valence (VB) bands with energy scales ranging from a few to hundreds of milli-electronvolts, involves only slight variations in crystal parameters. However, a major experimental challenge remains in uncovering the measurable effect of Berry curvature as an indicator of symmetry breaking under in situ successive modification of sublattice parameters.

In this study, we elucidate the intricate dynamics of ZrTe_5_ through comprehensive density functional theory (DFT) calculations integrated with ARPES analyses. Our research provides a direct avenue for investigating the Berry curvature physics underlying the temperature-induced TPT in ZrTe_5_ through low-energy photoexcitation involving geometric properties of the Bloch wavefunction intrinsic to the Dirac node. Our efforts culminate in the observation and analysis of symmetry-breaking-induced nonlinear current during the TPT process, driven by a nonzero Berry curvature dipole (BCD) that induces a nonlinear response upon the absorption of terahertz photons. These groundbreaking discoveries pave the way for exploiting the diverse topological states of ZrTe_5_ for practical applications, particularly in rapid imaging and remote sensing within the highly coveted terahertz spectrum.

## Results

### Theoretical and experimental demonstration of TPT

**Fig. 1.**
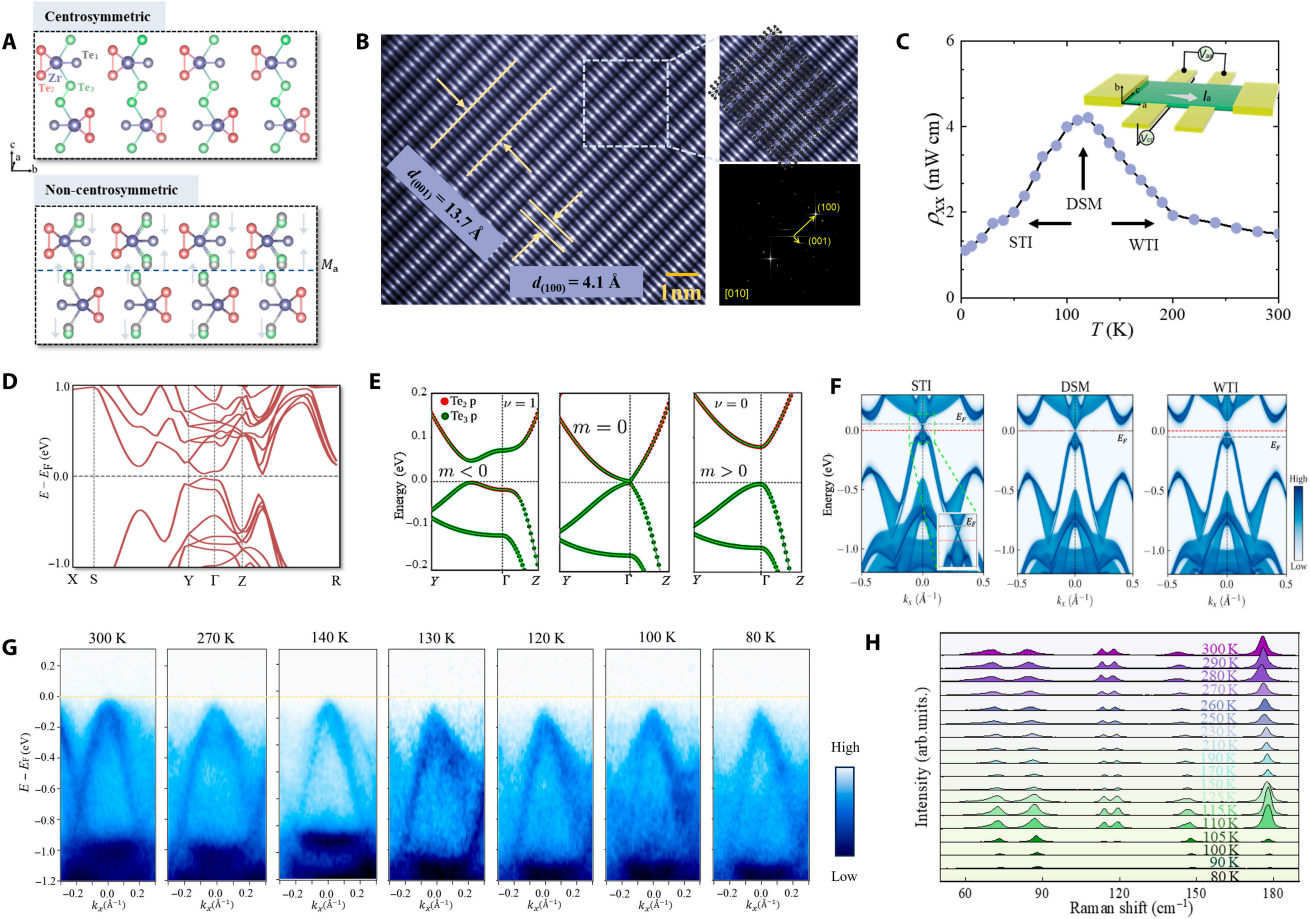
Crystal structural and topological phase characterization. (A) The top shows the centrosymmetric structure of ZrTe_5_, where the arrangement of Te atoms (Te_1_, Te_2_, and Te_3_) around Zr maintains inversion symmetry. The bottom displays the noncentrosymmetric structure, where the lack of inversion symmetry is evident across the mirror axis *M*_c_, indicating a shift in the arrangement of Te atoms along the *c* axis. Purple and black balls denote Zr and Te atoms, respectively. (B) High-resolution transmission electron microscopy image (left) shows the atomic lattice of ZrTe_5_ with exceptional crystalline fidelity. The scanning transmission electron microscopy image (top right) and the selected-area electron diffraction pattern (bottom right). (C) Temperature dependence of the longitudinal resistivity (*ρ_xx_*) in ZrTe_5_ nanosheets measured at zero magnetic field. (D) Calculated band structure of ZrTe_5_. The band structure indicates the presence of Dirac-like dispersion, suggesting that ZrTe_5_ may exhibit a semimetallic behavior. (E) Calculated band dispersion for bulk ZrTe_5_ along the *Y*–*Γ*–*Z* direction, showing topological phase transition (TPT) from the weak topological insulator (WTI) to Dirac semimetal (DSM) to strong topological insulator (STI) phase. The STI phase arises from the band inversion between Te_2_ p-orbitals and Te_3_ p-orbitals at the high-symmetry Γ point. (F) Theoretically calculated surface spectral function for 3 distinct phases. At the STI phase, there is a surface Dirac crossing as shown in the inset. (G) Angle-resolved photoelectron spectroscopy (ARPES) results measured at the range from 80 to 300 K. (H) Temperature dependence of the Raman intensity measured at the range from 77 to 300 K. The Raman spectrum of the obtained ZrTe_5_ nanosheets was derived from Raman spectroscopy (LabRAM HR800) with the laser wavelength of 532 nm through a 100× objective lens.

Figure 1A shows the orthorhombic crystal structure of ZrTe₅ with a *Cmcm* space group, consisting of 2 ZrTe_3_ chains connected by Te_2_ along the *c* axis. Each chain is composed of Zr atoms embedded in trigonal Te_1_–Te_3_ prisms along the *a* axis, and the layer stacking of ZrTe_5_ sheets along the *b* axis gives rise to a weak van der Waals (vdW) coupling. As shown in Fig. 1A, the ZrTe_5_ flakes break the inversion symmetry due to the staggered displacement of Te atoms along the *c* axis in the bottom part of Fig. 1A. The noncentrosymmetric topological materials in the field of optoelectronics offer significant advantages due to their enhanced nonlinear optical properties, which enriches the development of advanced photonic and optoelectronic devices [46]. To assess the structural quality of the chemical vapor transport (CVT)-synthesized ZrTe₅, we performed high-resolution transmission electron microscopy and aberration-corrected scanning transmission electron microscopy on exfoliated samples (Fig. 1B). The micrographs reveal clear lattice fringes with interplanar spacings of 13.7 and 4.1 Å, corresponding to the (001) and (100) planes of the orthorhombic structure. Selected-area electron diffraction patterns display sharp, well-defined spots, confirming high crystallinity. Figure 1C presents the temperature-dependent longitudinal resistivity (ρ_xx_) of our ZrTe₅ sample, revealing a characteristic peak at approximately 115 K that signals the TPT. This resistivity anomaly corresponds to the transition through the dynamic modulation of the Dirac polaron chemical potential that separates the STI phase at lower temperatures from the WTI phase at higher temperatures [44]. The observed transport behavior directly correlates with our temperature-dependent ARPES measurements and theoretical band structure calculations, confirming that the electronic structure undergoes a fundamental reorganization across this temperature range. This TPT is accompanied by a carrier-type inversion from n-type in the WTI regime to p-type in the STI regime, which fundamentally alters the Berry curvature distribution. These transitions are precipitated by pronounced spin–orbit interactions attributable to the presence of the heavy metal constituent Zr. The electronic band structures incorporating spin–orbit coupling effects are detailed in Methods, with the Brillouin zone (BZ) plotted in Fig. S1D.

Figure 1E shows the temperature-dependent topological transitions in ZrTe_5_ driven by Te orbital splitting, highlighting the splitting of Te atomic orbitals and the resulting electronic band structures for different topological states at varying temperatures. According to the Z_2_ topology invariant theory proposed by Kane and Mele [47], 3-dimensional (3D) time-reversal invariant TIs are characterized by 4 Z_2_ topological invariants (*ν*_0_, *ν*_1_, *ν*_2_, and *ν*_3_). Here, *ν*_0_ represents the strong topological index, while *ν*_1_, *ν*_2_, and *ν*_3_ are weak topological indices. STIs (*ν*_0_ = 1) possess topologically protected surface states on all surfaces, resulting from band inversion across the entire BZ. In contrast, WTIs (*ν*_0_ = 0 but at least one *ν*_i_ ≠ 0) exhibit protected surface states only on certain crystallographic surfaces, arising from band inversions on specific momentum planes. In ZrTe_5_, temperature-driven band structure evolution causes a transition between the WTI (0;110) and STI (1;110) phases, with dramatic consequences for transport and optical properties. Contrary to conventional thermodynamic phase transitions in compounds like VO_2_ and TaS_2_ [48], which typically involve substantial lattice rearrangements, TPTs often entail minimal or imperceptible adjustments in lattice constants. These transitions are rooted in modifications of the electronic topology rather than its lattice, specifically altering topological invariants. Due to the extreme sensitivity of the band topology to slight lattice or volume changes, the switching of symmetry in ZrTe_5_ serves as a control mechanism for inducing emergent nonlinear effects and concurrent TPT among different topological phases. The material maintains a stable WTI state at room temperature with p-type conductivity, which corresponds to the band structure at 300 K in Fig. S4. As temperature decreases, the layer spacing reduces and the bandgap narrows, transitioning to a DSM state at *T*_p_. Further cooling leads to the reopening of the bandgap and the Fermi level moving into the CB, resulting in an STI state with n-type conductivity. In the STI phase, spin-polarized surface states with Dirac crossings are evident (Fig. 1F). Temperature-dependent ARPES measurements conducted across a wide thermal range (80 to 300 K) reveal the systematic evolution of electronic structure through the TPT. As displayed in Fig. 1G, the ARPES intensity maps clearly demonstrate a temperature-driven band structure reconfiguration. With decreasing temperature from 300 to 80 K, we observe a progressive downshift of the VB maximum with respect to the Fermi level (demarcated by yellow dashed lines). Notably, the band dispersion gradually transitions from a parabolic to linear character at lower temperatures, manifesting the hallmark Dirac-like dispersion characteristic of the topological state. The measurements at intermediate temperatures (140, 130, and 120 K) capture the critical region of the TPT, where the electronic structure undergoes its most significant reorganization, providing direct spectroscopic evidence of the topological phase emergence. Figure 1H reveals the temperature-dependent Raman spectra of ZrTe_5_ nanosheets across 77 to 300 K, illustrating pronounced evolution of phonon dynamics near the TPT. Around 115 K, significant changes in peak intensity, linewidth, and frequency shifts emerge, corresponding to electron–phonon coupling reconstruction induced by the TPT. Notably, the characteristic peak at approximately 175 cm^−1^ exhibits nonmonotonic intensity enhancement and sharpening through the transition temperature, distinctly different from abrupt changes typical of conventional first-order structural transitions. Instead, we observe a progressive phonon response evolution reflecting subtle electron–lattice interactions during band inversion. The pronounced sensitivity of band topology to minute lattice offers exceptional tunability for terahertz detection, which involves ground and low-energy excited states that are characterized by the topological nature of Bloch wavefunctions.

### Switchable terahertz response across the TPT

**Fig. 2.**
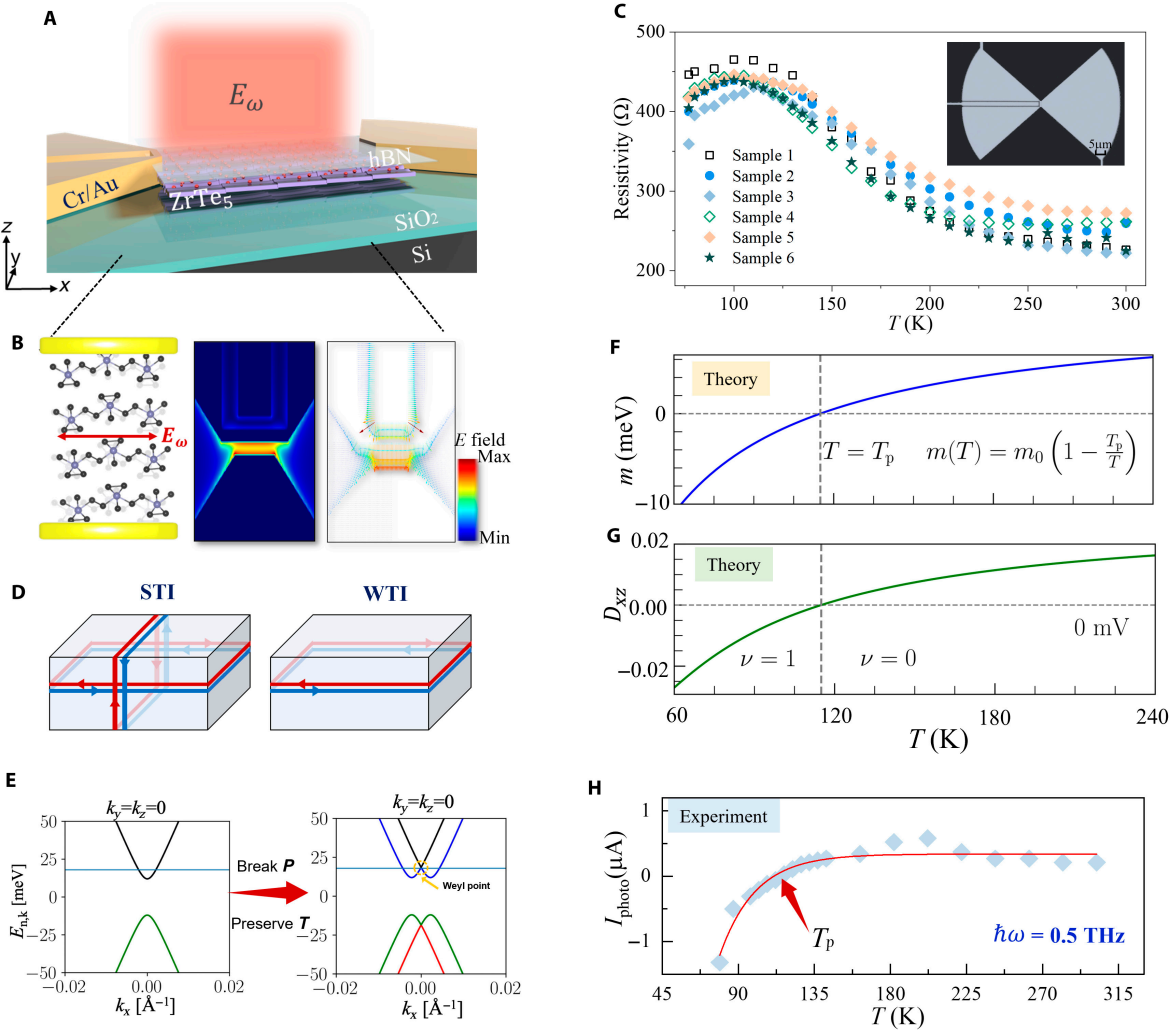
Device geometry and photocurrent analysis of the ZrTe_5_ device. (A) Experimental schematic (top) of the ZrTe_5_ device. Fan-shaped antenna simulations (bottom) showing the enhancement of the in-plane component of the electric field. (B) Detailed view of the fan-shaped antenna structure with the electric field (*E_ω_*) orientation on the left of the panel, which models the antenna as a perfect electrical conductor with a 50-μm radius, a 60° flare angle, and a 4-μm channel length on a SiO_2_/Si substrate to achieve a near-field enhancement factor of ~23. A vector plot of the electric field distribution, indicating the direction and intensity of the electric fields across the device in the right of the panel. (C) Two-terminal resistance as a function of temperature from 6 samples. (D) For STI, topological surface states distributing across all exterior facets. For WTI, topological surface states confining to the lateral surfaces of the material. (E) Demonstration of inversion symmetry breaking of ZrTe_5_. (F) A simple depiction of how the gap parameter, *m*, may be changing across the temperature-induced TPT (with *m*_0_ = 12 meV). (G) Theoretically calculated Berry curvature dipole (BCD) *d_xz_*, assuming the temperature variation of the bandgap *m* in (F). Due to the sign change of *d_xz_*, polarity of dc photocurrent changes across the TPT. (H) Experimentally measured temperature-dependent photocurrent under different conditions.

Figure 2A shows the architecture of a ZrTe_5_-based photodetector, which consists of a modified asymmetrical antenna, a multilayer of ZrTe_5_ synthesized via CVT, and a passivation layer of hexagonal boron nitride (h-BN). The ZrTe_5_ sheet is prepared by the mechanical exfoliation technique, and the device is meticulously crafted into a planar configuration with the fan-shaped antenna serving as 2 electrodes in Fig. 2B (see Methods for details). The impact of the fan-shaped antennae on device performance is thoroughly investigated through finite-difference time-domain (FDTD) simulations, as illustrated in Fig. 2B, where the local electric field distribution within the device channel reveals the fringe profile of the electric field lines from terahertz waves [49]. The enhanced in-plane electric field intensity at the active junction arises from a synergistic interplay of 3 mechanism-driven effects: pronounced near-field localization at the sharp fan-shaped edges, induced by the lightning rod effect; geometric wavefront engineering that concentrates terahertz radiation toward the central detection axis, and dimensionally optimized antenna structures resonantly coupled to the target terahertz spectral regime.

To enhance data accuracy, 6 sample sets were tested under uniform environmental conditions, with the results shown in Fig. 2C. Upon reaching the critical temperature of 115 K, the device resistance peaks at 450 Ω at the charge neutrality point (CNP), where the Fermi level (*E*_f_ ) coincides with the Dirac point. With further temperature increases (*T* > *T*_p_), resistance gradually decreases, stabilizing at 250 Ω as the Fermi level cuts the VB. Figure 2D effectively contrasts the behavior and distribution of topological surface states in the STI and WTI phases of ZrTe_5_. In the STI regime, robust topological surface states with suppressed backscattering dominate the transport, whereas in the WTI phase, the absence of protected surface states leads to bulk-dominated, less efficient carrier dynamics. Subsequent measurements are based on sample 1. Figure S8 shows the calculated conductivity (*σ*) of ZrTe_5_ devices, derived from source–drain current data in Fig. 2C, over a temperature range of 77 to 300 K in the dark. In this case, the conductivity as a function of temperature reaches a minimum when the Fermi level crosses the CNP at the TPT temperature, with a value of approximately 6.4 × 10^4^ S m^−1^. To uncover the Berry physics involving the ground states or low-energy states, we turn to the terahertz photoelectric process, and the broadband terahertz radiation, emanating from an array of VDI multiplier links, impinges on the ZrTe_5_ device (see Methods for details). The photoresponse of the device is meticulously quantified using a refined modulation technique, with the modulation frequency precisely calibrated to 1,000 ± 10 Hz. Concurrently, the photocurrent, *I*_photo_ (a critical parameter representing the transduction of terahertz light into electrical signals), is meticulously extracted via a sophisticated sampling method, employing a lock-in amplifier. It is observed that, within this configuration, the photocurrent response exhibits a polarity reversal as a function of temperature in Fig. 2H. Notably, the strength of photocurrent shows colossal sign-reversal behavior despite the minuscule change of resistance at temperatures below the transition point (*T*_p_). Due to the presence of both inversion and time-reversal symmetry, the bands are doubly degenerate for all *k* points. Based on the DFT calculation and its point group symmetry analysis, an effective 4 × 4 k.p model Hamiltonian around point Γ is given by:

$$H_{0}\left(k_{a},\;k_{b},\;k_{c}\right)=m1⊗\tau^{b}+\hbar \left(v_{a}k_{a}\sigma^{b}⊗\tau^{a}+v_{b}k_{b}\sigma^{a}⊗\tau^{a}+v_{c}k_{c}1⊗\tau^{c}\right)$$ (1)

Here, $\sigma^{\alpha }\;$and $\tau^{\beta }\;$represent Pauli matrices representing the spin and orbital degrees of freedom, respectively. The Hamiltonian preserves inversion, time reversal, and $M_{\mathrm{ab}}$, $M_{\mathrm{bc}}$, and $M_{\mathrm{ac}}$ mirror symmetries. In Eq. (1), *m* represents the mass of the Dirac fermion, which captures the bandgap at the Γ point, and $v_{i}$ and $k_{i}$ are the *i*th components of Fermi velocity and crystal momentum, respectively. The different parameters of the low-energy model are specified by *m* = 12 meV, $v_{a}$ = 9.7 × $10^{5}$ m/s, $v_{b}$ = 9.7 × $10^{4}$ m/s, and $v_{c}$ = 6.8 × 10^5^ m/s [50]. Owing to the presence of both inversion and time-reversal symmetry, all the bands are doubly degenerate.

The topology of the system is characterized by the sign of the bandgap, which is represented by the Dirac mass, whose magnitude is sensitive to the lattice parameters. By increasing the temperature, the system undergoes a transition from the STI to WTI phase through an intermediate DSM phase and is captured by the sign change of the parameter *m*, which changes from *m* < 0 in the STI phase with band inversion to *m* > 0 in the WTI phase. This is illustrated clearly in Fig. 2E via our DFT band structure calculations, where the WTI phase has no band inversion and the STI phase exhibits band inversion with an intermediate gapless Dirac phase. The temperature-induced phase transition in Fig. 2F can be modeled by defining an order parameter that varies with temperature as *m*(*T*) = *m*_0_ (1 − *T*_p_/*T*), where *T*_p_ denotes the transition temperature for which the band gap vanishes, indicating the DSM phase [36]. Additionally, bulk ZrTe_5_ breaks the inversion symmetry due to the staggered displacement of Te atoms along the *c* axis in Fig. 2E [51]. The structural distortion leads to the breakdown of $M_{\mathrm{ab}}$ and $M_{\mathrm{ac}}$ mirror symmetries, where inversion symmetry breaking can be modeled by the term:

$$H_{\mathrm{IB}}=\Delta 1⊗\tau^{a}+\xi \sigma^{a}⊗\tau^{c}$$ (2)

with ξ and ∆ representing the strength of $M_{\mathrm{ab}}$ and $M_{\mathrm{ac}}$ mirror symmetry breaking terms, respectively. Due to the breakdown of inversion symmetry with finite ∆ and ξ, we find that spin-split bands intersect at time-reversal invariant momentum point Γ, as shown in Fig. 2E. The BCD is a geometric property of the electronic band structure in momentum space, which plays a crucial role in nonlinear optical and electrical responses in materials with broken inversion symmetry. It quantifies the asymmetry of the Berry curvature (Ω) distribution and is defined as:

$$D_{\mathrm{xz}}=\int_{\mathrm{BZ}}\frac{dk}{{\left(2\pi \right)}^{3}}\Omega_{z}\left(k\right)\frac{\partial f\left(k\right)}{\partial k_{x}}$$ (3)

where *f*(**k**) is the Fermi–Dirac distribution function. Figure 2G shows the theoretical variation of the dipole moment (*d_xz_*) in ZrTe_5_, which transitions from negative to positive values as temperature increases from 60 to 240 K, corresponding to a topological phase change from a strong (*ν* = 1) to a weak (*ν* = 0) TI. The observed sign reversal in *d_xz_* aligns with the terahertz photocurrent in Fig. 2H and the Dirac mass sign change in Fig. 2F. The sign reversal of *d*_xz_ corresponds directly to the polarity switch of the terahertz response, providing a clear indicator of the TPT.

### BCD-induced nonlinear photocurrent in ZrTe_5_

**Fig. 3.**
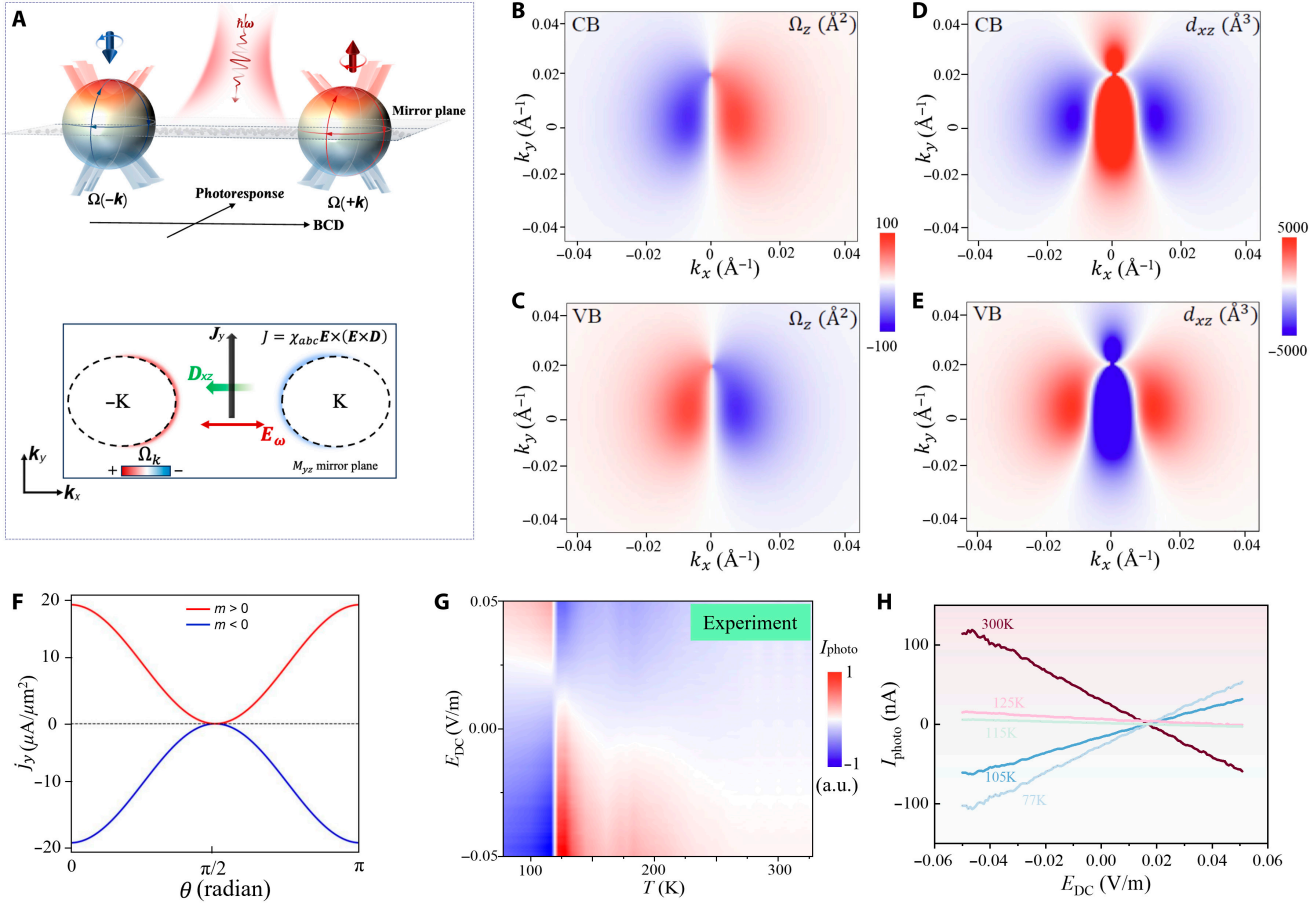
BCD-induced nonlinear photocurrent mechanism analysis. (A) Schematic figure for the Fermi surface that generates nonlinear second-order photocurrent. Low-energy-photon response due to the nonzero BCD. *Ω*(*k*) denotes the BCD vector. The left and right spheres represent electronic states with opposite momenta −*k* and +*k*, respectively, each influenced by the Berry curvature *Ω*. The sphere is colored to show variations in *Ω*, with blue indicating negative curvature and red indicating positive curvature. The arrows depict the direction of the Berry curvature vectors. The mirror plane indicates the noncentrosymmetric nature of the material. (B and C) Berry curvature of top valence band (VB) and bottom conduction band (CB) projected in *k_x_*, *k_y_* momentum space while *k_z_* = 0. (D and E) BCD density of the top VB and bottom CB projected in *k_x_*, *k_y_* momentum space while *k_z_* = 0. (F) Angular dependence of the terahertz photocurrent in ZrTe_5_. (G) Measured photocurrent as a function of temperature and dc electric field *E*_dc_. The color scale from blue to red represents the polarity and magnitude of the photocurrent. As temperature varies, the terahertz response exhibits a clear polarity switch, particularly around the topological phase transition temperature (~115 K). (H) Measured temperature-dependent photocurrent response in ZrTe_5_.

When a monochromatic light with electric field profile $E\left(t\right)=\left[E\left(\omega \right)e^{-\mathrm{i\omega t}}+E\left(-\omega \right)e^{\mathrm{i\omega t}}\right]$ is incident on a noncentrosymmetric material, we obtain a dc photoresponse in the second order of the electric field along a direction as:

$$j_{\alpha }=\sigma_{\alpha \beta \gamma }\left(0;\;\omega ,\;-\omega \right)E_{\beta }\left(\omega \right)E_{\gamma }\left(-\omega \right)$$ (4)

where $\sigma_{\alpha \beta \gamma }$(0, *ω*, −*ω*) represents second-order photoconductivity; *α*, *β*, and γ denote the coordinate directions; and ***E***(−*ω*) = E*(*ω*). The 2 prominent photocurrent contributions induced by linearly polarized light are the band geometric interband shift and the intraband BCD photocurrent. However, the shift photocurrent can be ruled out in our experiment, owing to the low energy of the incident photons in our experiment (0.41 to 2.5 meV), which is smaller than the ZrTe_5_ bandgap. Figure 3A illustrates the BCD mechanism underlying nonlinear terahertz photoresponse in noncentrosymmetric ZrTe₅. The upper panel depicts the fundamental asymmetry in Berry curvature distribution Ω(±**k**) across the mirror plane when the system is driven by terahertz radiation (*ħω*). This asymmetric distribution, represented by oppositely directed arrows (blue and red), is essential for generating nonzero BCD. The lower panel presents the microscopic origin of the nonlinear photocurrent generation: An incident terahertz electric field (*E*_ω_, red arrow) oriented along the *x* axis interacts with the Berry curvature gradient around the Fermi surface (depicted by the dotted circles at ±K). The coupling generates a BCD (*d*_xz_, green arrow) that produces a transverse photocurrent (*J*_y_) perpendicular to both the incident field and the mirror plane (*M*_yz_). The mechanism fundamentally differs from conventional photoresponse as it directly probes the topological characteristics of the electronic band structure, enabling the distinct photoresponse behaviors observed across the TPT in ZrTe_5_. The BCD-induced intraband dc photocurrent density is defined as [52–55]:

jα0=−2e3τℏ2Reτ1−iωτϵαβδDγδRe[(EβωEγ−ω]. (5)

Here, τ represents the electron scattering timescale, $\epsilon_{\alpha \beta \delta }$ denotes the antisymmetric tensor, and ω represents the frequency of incident light. In the low-frequency limit relevant to our experiment, ωτ→0, the factor $\;\frac{\tau }{1-\mathrm{i\omega \tau}}\to \tau$. In Eq. (5), $D_{\gamma \delta }$ denotes a rank 2 tensor, known as BCD, which is defined as:

$$D_{\gamma \delta }=-\int \left[\mathrm{dk}\right]\sum_{n}\frac{\partial f_{n}}{\partial \epsilon_{\mathrm{nk}}}v_{n}^{\gamma }\left(k\right)\Omega_{n}^{\delta }\left(k\right)\;$$ (6)

Here, $\Omega_{n}^{\delta }\left(k\right)$ is the band-resolved Berry curvature, $f_{n}$ denotes equilibrium Fermi–Dirac distribution, and $v_{n}^{\gamma }$ is the band velocity. Due to the presence of only $M_{\mathrm{yz}}$ mirror plane, only the $D_{\mathrm{xz}}$, $D_{\mathrm{zx}}$, $D_{\mathrm{xy}}$, and $D_{\mathrm{yx}}$ components of the BCD tensor are nonzero in the noncentrosymmetric ZrTe_5_ crystal. Thus, according to Eq. (5), there will be a dc photocurrent along the *y* axis, $j_{y}^{0}\sim D_{\mathrm{xz}}E_{x}E_{x}$ when the polarization axis of the incident light is along the *x* axis, or the *a*-crystalline axis.

Next, we present the calculated momentum-space distribution of the Berry curvature and BCD in ZrTe_5_. Figure 3B and C display the *z* component of the Berry curvature (Ω*_z_*) in units of square angstroms across the *k_x_*–*k_y_* plane for the CB and VB, respectively. The antisymmetric distribution of Ω*_z_* with respect to the *k_x_* = 0 line (positive/red on one side and negative/blue on the other) reflects the broken inversion symmetry while preserving mirror symmetry in ZrTe_5_. Figure 3D and E show the corresponding BCD component *d_xz_* in units of cubic angstroms, which measures the first moment of Berry curvature distribution. The interaction with terahertz light would likely lead to an asymmetric distribution of excited charge carriers due to the nonlinear response mediated by the BCD. This results in a directional photocurrent, as the dipoles *d_xz_* facilitate charge displacement predominantly along the *y* axis. In the experimental setup, the electric field polarization axis of the incident light is not perfectly aligned with the *x* axis because of the antenna, which generates electric field lines of electric dipole oscillation from metallic pads. Assuming the electric field profile $E=E\left(\text{cos}\theta ,\text{sin}\theta ,0\right)$, rotated by angle θ with respect to the *a*-crystal axis (as shown in Fig. 2B), we have both *x* and *y* components of the BCD-induced dc photocurrent as:

jx0=2e3τℏ2Reτ1−iωτDxzsinθcosθE2 (7)

jy0=−2e3τℏ2Reτ1−iωτDxzcos2θE2 (8)

Figure 3F effectively demonstrates that in noncentrosymmetric ZrTe_5_, a dc photocurrent $j_{y}^{0}$ is generated along the *y* axis when the polarization of the incident light is aligned with the *y* axis (*θ* = 90°), and the photocurrent vanishes when the polarization is along the *x* axis (*θ* = 0°). Additionally, the photocurrent reverses its polarity between the 2 topological phases characterized by *m* < 0 and *m* > 0 due to the sign change of *d_xz_* during the TPT. In conjunction with the TPT, a polarity reversal in the terahertz photocurrent was measured, as depicted in Fig. 3G, aligning closely with our theoretical predictions and confirming the behavior anticipated from the BCD in ZrTe_5_. The sign reversal of the terahertz photoresponse under positive *E*_dc_ in ZrTe₅ arises from a synergy between intrinsic symmetry breaking, topological band structure, and field-induced dynamic carrier redistribution. As previously established (Fig. 2G), the temperature-driven TPT in ZrTe_5_ shifts the Fermi level from the CB (STI phase) to the VB (WTI phase) near 115 K. This transition flips the polarity of the BCD *d_xz_*, which governs the nonlinear photocurrent via the relation *j*_α_ = *σ*_αβγ_ *E*_β_(ω) *E*_γ_(−ω). The sign change of *d_xz_* directly reflects the inversion of the effective mass parameter *m* across the Dirac point, serving as a hallmark of the topological phase boundary (Fig. 2F). While ZrTe_5_ lacks bulk inversion symmetry due to staggered Te atoms along the *c* axis, its low-energy physics is governed by a combined T–M symmetry (time-reversal × mirror) in the STI phase. Under an external *E*_dc_, this symmetry hierarchy is dynamically modified: Positive *E*_dc_ accelerates Bloch electrons, shifting their distribution toward regions of the BZ with opposite Berry curvature contributions (Fig. 3H). This displacement effectively reverses the net *d_xz_* in the CB-dominated regime (STI phase), triggering a sign switch in the terahertz response. As described by Morimoto and Nagaosa [16], the second-order nonlinear current follows *j_α_* = *σ_αβγ_ E_β_*(*ω*) *E_γ_*(−*ω*) *E*_dc_, where the second-order current depends on the momentum-space overlap of the Fermi distribution and Berry curvature gradients. Negative *E*_dc_ drives carriers into VB states where *d_xz_* remains positive (WTI phase), preserving the original response polarity. This asymmetry stems from the distinct spatial distributions of the Berry curvature in CB and VB bands. Near the Dirac point (115 K), the Fermi surface transitions between these regimes, amplifying the sensitivity to *E*_dc_-induced shifts. The CB and VB exhibit asymmetric dispersion along the *k_z_* direction. Positive *E*_dc_ preferentially probes CB regions with the reversed Berry curvature, while negative *E*_dc_ confines carriers to VB pockets where *d_xz_* > 0 persists. Although static inversion symmetry is absent, T–M symmetry stabilizes the STI phase. Positive *E*_dc_ introduces a momentum-dependent perturbation that explicitly breaks *M_z_*, allowing interband coupling between regions of opposing Berry curvature. Negative *E*_dc_ instead reinforces *M_z_*-compatible carrier localization, suppressing such coupling. The ability to switch the polarity of the terahertz response by tuning the temperature and applying an external dc field is a notable feature. While the overall photocurrent magnitude is inevitably influenced by temperature-dependent transport parameters, the observed polarity reversal across the phase boundary (Fig. 3H) provides critical insight into the dominant generation mechanism. Such a sign inversion is difficult to reconcile with conventional photothermal effects, which are typically polarity-definite for a given device geometry. Instead, it strongly points to a mechanism intrinsic to the energy topology, such as the nonlinear photocurrent generated by the BCD. The BCD is predicted to invert its sign across the WTI-to-STI transition, naturally explaining our central observation. Therefore, we propose that the observed photoresponse is primarily a topological effect dominated by the BCD, while its magnitude is co-modulated by factors including the density of states (both surface and bulk) and thermally activated transport processes.

### Terahertz detection performance characterization

**Fig. 4.**
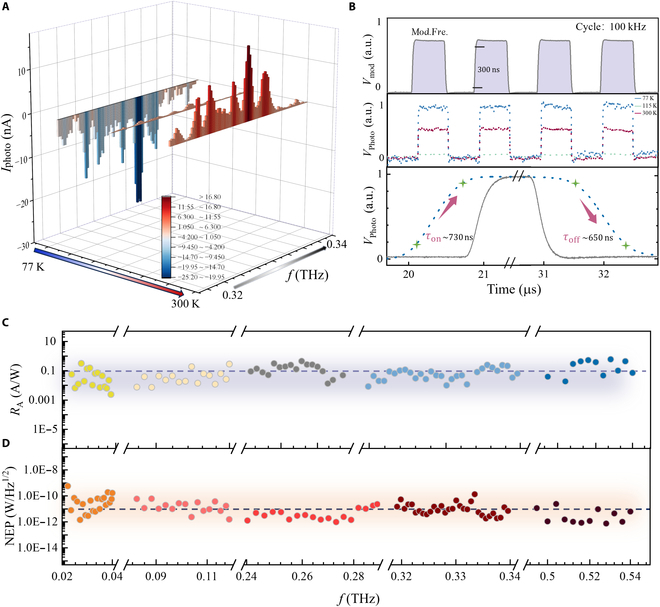
Terahertz photodetection performance of the photodetector. (A) Photocurrent at different operating temperatures and zero-bias voltage. (B) Time-resolved photoresponse of the device without any external field under 0.3-THz illumination at a modulation frequency of 100 kHz. Estimated rise and fall times of 630 and 750 ns, respectively (solid blue line). Frequency-dependent responsivity in (C) and noise equivalent power (NEP) in (D) based on sample 1. Terahertz measurements using the ZrTe_5_-based detector at zero-bias operation. The maximum measured frequency is 0.54 THz.

Figure 4A presents a comprehensive 3D visualization of the measured photocurrent response in ZrTe_5_ devices as a function of both radiation frequency (0.32 to 0.34 THz) and temperature (77 to 300 K). The waterfall plot elegantly captures the dramatic evolution of the terahertz photoresponse across the TPT. At 77 K, the device exhibits a pronounced negative photocurrent (reaching approximately −30 nA at peak frequencies), characteristic of the STI phase where CB states with negative BCD dominate the response. As the temperature increases toward the TPT point (~115 K), the photocurrent magnitude systematically decreases and undergoes a complete polarity reversal, transitioning to positive values at 300 K where the WTI phase prevails. Figure 4B characterizes the temporal response dynamics of our ZrTe_5_-based terahertz detector under pulsed terahertz excitation. The upper panel displays a 300-ns electrical modulation pulse (Mod. Freq.) applied at a repetition rate of 100 kHz, monitored using a high-bandwidth oscilloscope. The middle panel reveals the temperature-dependent photocurrent response (*V*_photo_) at 3 representative temperatures (77, 115, and 300 K), demonstrating the critical influence of thermal conditions on detector performance. At 77 K, the photoresponse exhibits a significantly enhanced signal amplitude and a superior signal-to-noise ratio due to suppressed phonon scattering and reduced thermal carrier excitation, which preserves the topologically protected carrier transport characteristics. As temperature increases to 115 K (near the TPT point), the photoresponse becomes attenuated with increased baseline fluctuations, reflecting the enhanced carrier–phonon interactions at the critical transition temperature. At 300 K, thermal broadening severely degrades signal quality, consistent with the diminished BCD contribution at elevated temperatures. The bottom panel presents a high-resolution temporal analysis of the 77-K photoresponse, revealing critical optoelectronic transient characteristics. The waveform analysis yields rise and fall time constants of *τ*_on_ ≈ 730 ns and *τ*_off_ ≈ 650 ns, respectively. These temporal parameters translate to corresponding frequency-domain bandwidths of approximately 218 kHz (1/2*πτ*_on_) and 245 kHz (1/2*πτ*_off_), establishing the detector’s operational bandwidth for high-frequency sensing applications. The asymmetry between rise and fall times (τ_on_, τ_off_) suggests distinct physical mechanisms governing carrier generation versus relaxation processes, likely attributed to the interplay between photoexcited carrier dynamics and the Berry curvature in the topological bands of ZrTe_5_. Figure 4C shows the measured frequency-dependent responsivity with broadband excitation from the VDI frequency multiplier link, ranging from 0.02 to 0.54 THz, showing a prevalent trend of responsivity values clustering around the vicinity of 0.1 A/W. The broadband response is particularly noteworthy compared to resonant or narrowband detectors that typically function effectively only within specific frequency windows. The observed frequency dependence of the photoresponse can be understood as a convolution of 2 primary factors: (a) the linear absorption coefficient of ZrTe_5_, which governs the overall envelope of the response spectrum, and (b) the intrinsic frequency dispersion of the nonlinear topological mechanism itself, namely, the BCD response. While the broad decay of the signal toward higher frequencies is consistent with general material absorption, the nontrivial spectral features and deviations from a simple absorption profile strongly suggest that the BCD response is also frequency-dependent. Therefore, we interpret the spectral response as a hybrid phenomenon where linear absorption dictates the broad shape, while the BCD mechanism governs the fundamental nonlinear character and contributes its own intrinsic dispersion*.* To further evaluate photodetection sensitivity, NEP is calculated by measuring the noise spectral density (*i*_n_) without light illumination at different modulation frequencies (Fig. S10) and the current responsivity (NEP = *i*_n_*/R*_i_) of the detector for different frequencies, where NEP reaches a mean level of approximately 20 pW Hz^−1/2^ in the range from 0.02 to 0.54 THz at ambient temperature in Fig. 4D. The performance represents a significant advancement over conventional commercial terahertz detectors. For comparison, standard commercial room-temperature terahertz detectors typically exhibit NEP values of 1 × 10^−9^ W/Hz^1/2^ in similar frequency ranges. Our achieved NEP is therefore approximately 2 orders of magnitude lower than these traditional thermal photodetectors operating at room temperature while maintaining broadband detection capability across the measured terahertz range [56]. The detection mechanism maintains high sensitivity at room temperature without cryogenic cooling requirements, which would otherwise introduce additional noise sources and system complexity. While the strongest photoresponse is observed in the STI phase at cryogenic temperatures, we note that the ZrTe_5_ detector maintains competitive responsivity and ultralow NEP even at room temperature. This performance originates from residual nonlinear responses allowed by the underlying band topology in the WTI phase, highlighting the potential for topologically enabled terahertz sensing beyond the cryogenic limit. Terahertz nondestructive imaging has become an indispensable tool in a variety of scientific and practical fields due to its ability to perform deep material analysis without causing damage. The experimental setup of terahertz imaging in Fig. S12 displays the terahertz transmission imaging of unidentified liquids, 3D-printed polyvinyl chloride (PVC) handguns, and knives in envelopes. Experimental results in Fig. S12, including the analysis of unidentified liquids and detection of concealed 3D-printed weapons, highlight the precise imaging capabilities and potential for improving security protocols. The integration of ZrTe_5_-based terahertz detectors into array configurations holds promising potential for advancing future nondestructive imaging applications, offering enhanced sensitivity, resolution, and real-time imaging capabilities for diverse fields such as security screening, medical diagnostics, and materials characterization.

## Discussion

Our study represents a significant advancement in terahertz technology by leveraging the geometric and topological properties of Bloch quasiparticles in TI ZrTe_5_. We demonstrate a robust terahertz photocurrent, modulated by TPTs, with a remarkable response speed (<1 μs) and a high signal-to-noise ratio, surpassing traditional technologies. Theoretical findings corroborate the microscopic origin of photocurrent switching, which is associated with the sign change of Dirac mass (*m*) during the TPT from strong TI to weak TI. The development of a high-quality ZrTe_5_ crystal and its integration into a vdW heterojunction significantly boosts device responsivity to 0.4 A/W and reduces NEP to an impressive 5.6 pW/Hz^1/2^. Although the TPT is induced statically via temperature, its impact on the terahertz photoresponse is profound: The topological reconfiguration of the band structure alters the BCD, enabling a polarity-reversible, symmetry-governed nonlinear response to low-energy terahertz excitation. This establishes TPT as a powerful control axis for terahertz detection engineering.

## Methods

### Material growth of ZrTe_5_

High-quality ZrTe_5_ single crystals are synthesized by the CVT method. Firstly, Zr (foil, 99.2%, Sigma-Aldrich) and Te (powder, 99.8%, Alfa Aesar) were put into a quartz tube in a corresponding stoichiometric ratio (1:5). The quartz tube was sealed under a high vacuum of 1.0 × 10^−4^ Pa. Next, the quartz tube was placed in a quartz tube furnace, heated to 500 °C, and maintained for 24 h, then quickly lowered to 500 °C, and slowly lowered to 400 °C at a rate of 1 °C/h. Finally, the temperature was lowered to 300 °C over 12 h, and after returning to room temperature, ZrTe_5_ polycrystalline powder was obtained. Secondly, the ZrTe_5_ polycrystalline powder and iodine were enclosed in an evacuated quartz tube as the precursor for the growth. Then, the quartz tube was placed horizontally in the 2-zone quartz tube furnace between 550 °C (hot zone, source materials) and 450 °C (cold zone, sink) for 2 weeks. When the temperature of the quartz tube cooled down to ambient temperature, high-quality ZrTe_5_ single crystals were obtained.

### Device fabrication

The ZrTe_5_ flakes were exfoliated from a bulk crystal using scotch tape and then transferred onto a high-resistance intrinsic silicon substrate (*ρ* > 20,000 Ω·cm) with a thermally grown oxide layer SiO_2_ (285 nm). Subsequently, the source and drain electrodes of ZrTe_5_ terahertz devices were defined by EBL (JEOL JBX 5500). The electron beam evaporator (Ulvac Ei-5Z) was then used to deposit the Cr/Au (10/70 nm) film, after which a lift-off process was performed to obtain the desired electrodes. The heterojunction device was fabricated as follows: Few-layer graphene was obtained by the standard mechanical exfoliation method, and the layered graphene was transferred from blue tape to polydimethylsiloxane (PDMS). Then, the layered graphene was transferred onto ZrTe_5_ by dry transfer under the optical microscope of a precision transfer platform (meta test, E1-T) to complete the preparation of the heterojunction. Electrode structures were formed on the substrate by electron beam exposure, then 70-nm Cr/Au contacts were deposited by electron beam evaporation, and finally ZrTe_5_-graphene heterostructure terahertz detectors were formed by the lift-off technique.

### Photoresponse measurements

The electrical properties of the devices were measured by a semiconductor parameter analyzer (B2912A) in variable voltage mode. For photoresponse measurements, data were acquired using a custom-built optical setup. The output signal of a stabilized microwave source (Agilent E8257D) at 0.04 THz, providing low-energy photons, was connected to a frequency multiplier (VDI WR 9.0, VDI WR 2.8) and lock-in (SR830) amplification technique. Electromagnetic waves of 0.34 and 0.5 THz were generated by a customized VDI frequency multiplier. The terahertz wave was transistor–transistor logic (TTL) modulated in its amplitude (electronically chopped) with a 1-kHz square-wave signal to facilitate the use of lock-in techniques in the presence of dc offset and 1/f noise. A commercially calibrated photoconductive terahertz receiver (TK100) was used. For temperature-dependent measurements, the device was mounted in a Lake Shore CRX-6.5K closed-cycle cryostat, with the temperature actively regulated by a Lake Shore Model 336 Temperature Controller to within 10 mK after a 15-min stabilization period at each set point. The sample was mounted on a gold-plated cold finger using thermally conductive silver paste to ensure uniform thermal contact. Before each data acquisition, the system was allowed to reach thermal equilibrium for at least 5 min to ensure temperature stability. The responsivity, *R*_v_, was extracted from the measured *I*_ph_ as *R*_v_ = *I*_ph_*R*/(*PS*), where *P* is the power density and *S* is the diffraction-limited area *S* = *S*_λ_ = *λ*^2^/4*π*. The noise-equivalent power, NEP, was extracted from the formula NEP = *ν*_n_/*R*_v_, where *ν*_n_ is the root mean square of the noise voltage and *R*_v_ is the voltage responsivity. The theoretical NEP was extracted from the formula NEP = (4*k*_B_*Tr*)^0.5^/*R*_v_, where *k*_B_ is Boltzmann’s constant, *T* is the temperature of the detectors, and *r* is the resistance of the device. All measurements were performed under ambient conditions. Noise measurements were performed at room temperature in a Lakeshore cryogenic probe station with micromanipulation probes.

## Data Availability

The data that support the findings of this study are available from the corresponding authors upon reasonable request.

## Supplementary Materials

Supplementary 1 Sections S1 to S5 Figs. S1 to S13
