# Supplementary material for: Dynamic Polarization Control of Nonlinear Terahertz Photoresponse via Topological Phase Transitions
Source: Research (Wash D C). 2025 Sep 25;8:0899. doi: 10.34133/research.0899 (PMC12460995; doi:10.34133/research.0899)
Supplement: Supplementary 1 — Sections S1 to S5 Figs. S1 to S13 [file research.0899.f1.docx]

Supplementary Information

**Dynamic Polarization Control of Nonlinear Terahertz Photoresponse via** **Topological Phase Transitions**

Libo Zhang^1^^†^, Xuyang Lv^2†^, Dong Zhuo^3†^, Debasis Dutta^4^, Liu Yang^3^, Raihan Ahammed^4^, Atasi Chakraborty^4,5^, Dong Wang^3^, Zhen Hu^2^, Mengjie Jiang^1^, Kaixuan Zhang^1^, Li Han^6^, Amit Agarwal^5*^, Kai Zhang^3^,  Lin Wang^2*^, Xiaoshuang Chen^1,2*^

^1^College of Physics and Optoelectronic Engineering, Hangzhou Institute for Advanced Study, University of Chinese Academy of Sciences, No. 1, Sub-Lane Xiangshan, Xihu District, Hangzhou 310024, China.

^2^State Key Laboratory of Infrared Physics, Shanghai Institute of Technical Physics, Chinese Academy of Sciences, 500 Yu-Tian Road, Shanghai 200083, China.

^3^CAS Key Laboratory of Nanophotonic Materials and Devices & Key Laboratory of Nanodevices and Applications, i-Lab, Suzhou Institute of Nano-Tech and Nano-Bionics, Chinese Academy of Sciences, Suzhou 215123, P. R. China.

^4^ Department of Physics, Indian Institute of Technology Kanpur, Kanpur 208016, India.

^5^ Institute für Physik, Johannes-Gutenberg-Universität Mainz, D-55099 Mainz, Germany.

^6^ College of Optical and Electronic Technology, China Jiliang University, Hangzhou 310018, China;

**Supplementary section S1 DFT calculations**

First-principles density functional theory (DFT) calculations are carried out within a plane-wave basis for a realistic description of the problem. The structural optimization, as well as the influence of spin-orbit coupling (SOC) on the electronic structure are investigated using the projector augmented wave (PAW) potentials implemented within the Vienna Ab initio simulation package (VASP). In the plane-wave calculations, the wave functions in the plane-wave basis are expanded with a kinetic-energy cutoff of 400 eV. For self-consistent calculations with a plane-wave basis, the k-point meshes are chosen to be 12 × 6 × 6. Calculations are carried out with a chosen exchange-correlation functional of the generalized gradient approximation (GGA) within the framework of Perdew-Burke-Ernzerhof (PBE). To accommodate the effect of the layered structure of ZrTe_5_ orthorhombic crystal, we have included non-local vdW corrected optB86b-vdw functional within GGA approximation implemented in VASP code. The SOC is included in the calculations as a second variational form to the original Hamiltonian. To account for the temperature dependence on the physical property of the systems, we have hydrostatically changed the volume of the unit cell. For each perturbed structure unit cell to get the minimum energy structure, we have considered optimization through symmetry-protected ionic relaxation until the Hellmann Feynman forces per atom are less than 0.01 eV/Å.To calculate the surface spectral function for finite geometry slabs of ZrTe_5_, we construct the tight-binding model Hamiltonian by deploying atom-centered Wannier functions within the VASP2WANNIER90 codes. Utilizing the obtained tight-binding model, we calculate the surface spectral function using the iterative Green’s function method, as implemented in the WannierTools package. We modeled the effect of temperature on the material by varying the interlayer spacing. To reveal the reason for the origin of TPT, we execute the evolution of the band topology under temperature in Fig. S4 (bulk and surface states) and in Fig. S6 (surface states). Figure S4d provides a detailed view of the band structures before, during, and after the topological phase transition (TPT). The left plot shows the energy dispersion in the strong topological insulator (STI) phase, characterized by a clear energy gap. The middle plot represents the Dirac semimetal (DSM) phase at the transition point, where the bands close at the Dirac point. The right plot illustrates the weak topological insulator (WTI) phase post-transition, where band inversion and reopening occur, consistent with the theoretical predictions of topological phase transitions. These findings are corroborated by recent studies in the literature^1^, which have reported similar SOC-induced band modifications and topological phase transitions in ZrTe_5_ and related materials. Our DFT calculations thus provide a comprehensive understanding of the topological nature of ZrTe_5_, paving the way for further experimental and theoretical explorations in topological quantum materials.

**Supplementary section S2 ARPES**

To accurately identify the characteristic signatures of a topological phase transition (TPT) in ZrTe_5_, we utilized angle-resolved photoelectron spectroscopy (ARPES), as shown in Fig. S6b. The ARPES data reveal both surface and bulk states at the top of the valence band. The ARPES data clearly demonstrate that the top of the valence band is located at the Γ point, consistent with DFT calculations. As the temperature falls below *T*_p_, the Fermi surface enters the conduction band, as shown in ARPES measurements at liquid nitrogen temperature. This observation strongly supports the occurrence of a phase transition. In Fig. S6b, the ARPES data reveal both surface and bulk states at the top of the valence band. At higher temperatures (above the transition temperature *T*_p_), the Fermi level is located within the valence band, indicating that ZrTe_5_ exhibits a semimetallic behavior. The data clearly show that the top of the valence band is situated at the Γ point, consistent with density functional theory (DFT) calculations. This alignment with theoretical predictions underscores the reliability of our ARPES measurements. As the temperature decreases and crosses *T*_p_, significant changes are observed in the band structure. The ARPES spectra demonstrate a movement of the Fermi level into the conduction band, signifying the onset of the topological phase transition. This shift indicates that the material transitions from a semimetal to a topologically non-trivial phase, characterized by the emergence of topologically protected surface states. The ARPES data at liquid nitrogen temperature (approximately 77 K) further confirm this transition. At this lower temperature, the Fermi surface distinctly enters the conduction band, providing robust evidence of the phase transition. This observation is consistent with recent studies on topological materials, where similar temperature-dependent behavior has been reported^2-4^. To further elucidate the nature of the topological states, we analyzed the surface spectral function using the iterative Green’s function method, implemented in the WannierTools package. This analysis revealed the presence of Dirac-like surface states that persist below *T*_p_, confirming the topological nature of ZrTe_5_ in its low-temperature phase in Fig. S6a-c. ARPES measurements, supported by DFT calculations and surface spectral function analysis, provide compelling evidence of a topological phase transition in ZrTe_5_. The clear shift of the Fermi level from the valence band to the conduction band as the temperature decreases, along with the emergence of topologically protected surface states, highlights the material’s potential for applications in topological quantum computing and other advanced electronic devices. The Supplementary Fig. S4d illustrates the corresponding 3D Brillouin zone with high-symmetry points of ZrTe_5_ with projected surface Brillouin zone onto (010) surface. A further rise in temperature results in the reformation of the bandgap, thus reinstating a weak topological insulating phase. The temperature-dependent evolution from an STI to a WTI state, traversing through a gapless DSM phase, aligns with theoretical prognostications previously posited^5^.

**Supplementary section S3 The evidence of phase transition in ZrTe_5_**

The most persuasive explanation for the maximum resistance observed in ZrTe_5_ at the semimetal state during the topological phase transition relates to the temperature-induced Lifshitz transition. This transition involves a change in the Fermi surface topology, which significantly impacts the material's electronic properties and resistivity. Their high-resolution laser-based angle-resolved photoemission measurements revealed the temperature evolution of the electronic structure, supporting the notion that the resistivity anomaly in ZrTe_5_ is a direct consequence of the transition.

Furthermore, Raman spectra, shown in Fig. S2, is also performed out to conﬁrm the chemical structure of multilayer ZrTe_5_ ﬂake, and six prominent Raman peaks B_2g_^1^, B_2g_^2^, A_g_^1^, A_g_^2^, A_g_^3^ and A_g_^4^ at 72, 86, 116, 121, 148 and 182 cm^−1^ conform well with theoretically documented results at 300K. Increasing temperature from 77 to 300K results in the progressive red-shift, broadening, and intensity reduction of all peaks. Each horizontal line represents the Raman signal intensity (in arbitrary units, a.u.) as a function of the Raman shift, for a specific temperature labeled on the right side of the graph. As the temperature decreases from 300 K to 77 K, several key changes in the Raman spectra can be observed: The position and intensity of the Raman peaks vary with temperature, indicating changes in the phonon modes of ZrTe_5_. At higher temperatures (300 K to 250 K), the peaks are broader and less defined. As the temperature decreases (250 K to 77 K), the peaks become sharper and more distinct, showing clear shifts in their positions. Notable peaks are observed around specific Raman shifts, and these peaks become more prominent and defined at lower temperatures, suggesting enhanced phonon coherence and reduced thermal broadening. This temperature-dependent Raman analysis provides insights into the lattice dynamics and phonon interactions in ZrTe_5_, highlighting how temperature affects its vibrational properties.

**Supplementary section S4 Bandgap Variation of ZrTe_5_ Nanosheets**

Supplementary Fig. S5 shows the temperature-dependent bandgap variation (ΔE_g_) of ZrTe_5_ nanosheets, highlighting the transition from n-doping to p-doping regimes. At lower temperatures (below ~135 K), ZrTe_5_ exhibits n-type doping with a decreasing bandgap. The minimum bandgap near the transition temperature (~135 K) corresponds to the Dirac semimetal phase, a key feature of the topological phase transition (TPT). As the temperature increases, the bandgap reopens, indicating a transition to p-type doping and the emergence of a topologically non-trivial phase with protected surface states. This temperature-dependent bandgap behavior aligns with recent findings in the literature. Studies have shown that the bandgap modulation in ZrTe_5_ is closely linked to its topological properties and doping effects. The presence of a Dirac semimetal phase at the transition point is a hallmark of ZrTe_5_ unique electronic structure, which is tunable via temperature and doping. The observed bandgap reopening at higher temperatures further supports the material’s potential for applications in topological quantum devices and thermoelectric materials.

**Supplementary section S5 BCD-induced nonlinear photocurrent in bulk ZrTe_5_**

Fig. S7 c-d represent the dipole density components d_xz_ for the VB and CB. The distribution is more concentrated near the center, showing intense blue and red lobes along the k_x axis, reflecting the strong anisotropy in the Berry curvature dipole. Supplementary Fig. S7 illustrates the Berry curvature (BC) and Berry curvature dipole (BCD) density for the top valence band (VB) and bottom conduction band (CB) of ZrTe_5_, projected in the k_x_, k_y_ momentum-space at k_z_ = 0. These visualizations are critical in understanding the nonlinear optical properties of ZrTe_5_, particularly in the context of BCD-induced nonlinear photocurrents. The BC for the VB and CB shows distinct regions of positive and negative curvature, indicating a non-trivial topology in Fig. S7 a-b. In the valence band (Fig. S7 a), there is a notable red-blue contrast along the k_x_ = 0 axis, demonstrating the presence of Berry curvature hotspots. Similarly, in the conduction band (Fig. S7b), the BC distribution is mirrored, highlighting the symmetry between the VB and CB near the Dirac point. Fig. S7 c-d represent the dipole density components d_xz_ for the VB and CB. The distribution is more concentrated near the center, showing intense blue and red lobes along the k_x_ axis, reflecting the strong anisotropy in the Berry curvature dipole. The d_yz_ components show a similar anisotropic distribution but along the k_y_ axis, with significant regions of red and blue indicating dipole density variations perpendicular to the k_x_ axis in Fig. S7 e-f.

The Berry curvature dipole (BCD) plays a crucial role in the generation of second-order nonlinear photocurrents in non-centrosymmetric materials. In ZrTe_5_, the asymmetry in the Berry curvature dipole density, as depicted in the figures, is directly related to the strength and directionality of the induced photocurrent. The nonlinear photocurrent is a second-order response facilitated by the Berry curvature dipole. This is because the BCD acts as an effective electric field in momentum space, driving charge carriers in a non-centrosymmetric manner^6^. ZrTe_5_, with its distinct BC and BCD distributions, shows potential for high-efficiency photodetection and photovoltaic applications. The large Berry curvature and its dipole indicate strong interaction with light, leading to substantial photocurrent generation even in the absence of external fields. The symmetry properties of ZrTe_5_ are crucial in determining the orientation and magnitude of the BCD. The figures indicate that the BCD is highly anisotropic, which suggests directional control over the photocurrent can be achieved by engineering the crystal orientation^7^.

The corresponding photo-conductivity $\sigma_{\alpha\beta\gamma}^{\mathrm{BCD}}$ relating the Non-linear current is given by：

$$\sigma_{\alpha\beta\gamma}^{\mathrm{BCD}}={\frac{e^{3}\tau}{\hbar^{2}}\epsilon}_{\alpha\beta\delta}D_{\gamma\delta}$$

Here, −e is the electronic charge, $\epsilon_{\alpha\beta\delta}$ is the anti-symmetric Levi-Civita tensor, τ is the scattering time and $D_{\gamma\delta}$ is the BCD.

The BCD pseudovector can be defined as **Λ** = (Λ_x_, Λ_y_, 0); Λ_x_ = D_yz_, Λ_y_ = D_xz_.

As, **j**^2ω^ ∝ **E** × (**Λ** × **E**), if the electric field **E** is subjected by angle θ to the x-axis in x-y plane, then E_x_, E_y_ = E (cos θ,sin θ), which results in

$\frac{j_{\theta}^{2\omega}}{E^{2}}\propto\hat{i}$(Λ_y_ sin θ cos θ + Λx sin^2^θ)- $\hat{j}$(Λ_y_ cos^2^θ + Λ_x_ sin θ cos θ),

∝ −(Λ_y_ cos θ + Λ_x_ sin θ)(− $\hat{i}$sin θ + $\hat{j}$ cos θ) ,

∝ −Λ$\hat{\theta}$

The angular dependence of $j_{x}^{\mathrm{BCD}}$and$j_{y}^{\mathrm{BCD}}$can be expressed as,

$j_{x}^{\mathrm{BCD}}$ = $\frac{e^{3}\tau}{\hbar^{2}}$(Λ_y_ sinθ cosθ + Λ_x_ sin^2^θ) | E_0_ |^2^

$j_{y}^{\mathrm{BCD}}$ = −$\frac{e^{3}\tau}{\hbar^{2}}$(Λ_x_ sinθ cosθ + Λ_y_ cos^2^θ) |E_0_|^2^

These symmetry operations can be visualized in Fig. 2g-h and **Supplementary Fig. S7-8**. Under mirror symmetry $\mathcal{M}$_yz_, Berry curvature (BC) and BC dipole transform as Ω_z_ (**k**) = Ω_z_ (-k_x_, k_y_, k_z_), D_xz_ (**k**) = D_xz_(-k_x_, k_y_, k_z_), D_yz_ (**k**) = -D_yz_(-k_x_, k_y_, k_z_). Under time-reversal symmetry ($T$), it transforms as Ω_z_ (**k**) = -Ω_z_ (-k_x_, -k_y_, -k_z_), D_xz_ (**k**) = D_xz_(-k_x_, -k_y_, -k_z_), D_yz_ (**k**) = D_yz_(-k_x_, -k_y_, -k_z_). So, under combined action of T and $\mathcal{M}$_yz_, berry curvature (BC) and BC dipole transform as Ω_z_ (**k**) = -Ω_z_ (k_x_, -k_y_, -k_z_), D_xz_ (**k**) = D_xz_(k_x_, -k_y_, -k_z_), D_yz_ (**k**) = D_yz_(k_x_, -k_y_, -k_z_). The BCD-induced photocurrent response of bulk ZrTe_5_ is depicted in Fig. 3a. Berry curvature dipole (BCD) and BCD-induced responses of bulk ZrTe_5_ change sign when switching the sign of the Dirac mass m. Only the in-plane components $\sigma_{\mathrm{yxx}}^{\mathrm{BCD}}$ and $\sigma_{\mathrm{xyx}}^{\mathrm{BCD}}$ survive for the non-zero BCD component D_xz_. The other in-plane components $\sigma_{\mathrm{xyy}}^{\mathrm{BCD}}$ and $\sigma_{\mathrm{yxy}}^{\mathrm{BCD}}$ are found to be zero for the vanishing BCD component D_yz_.


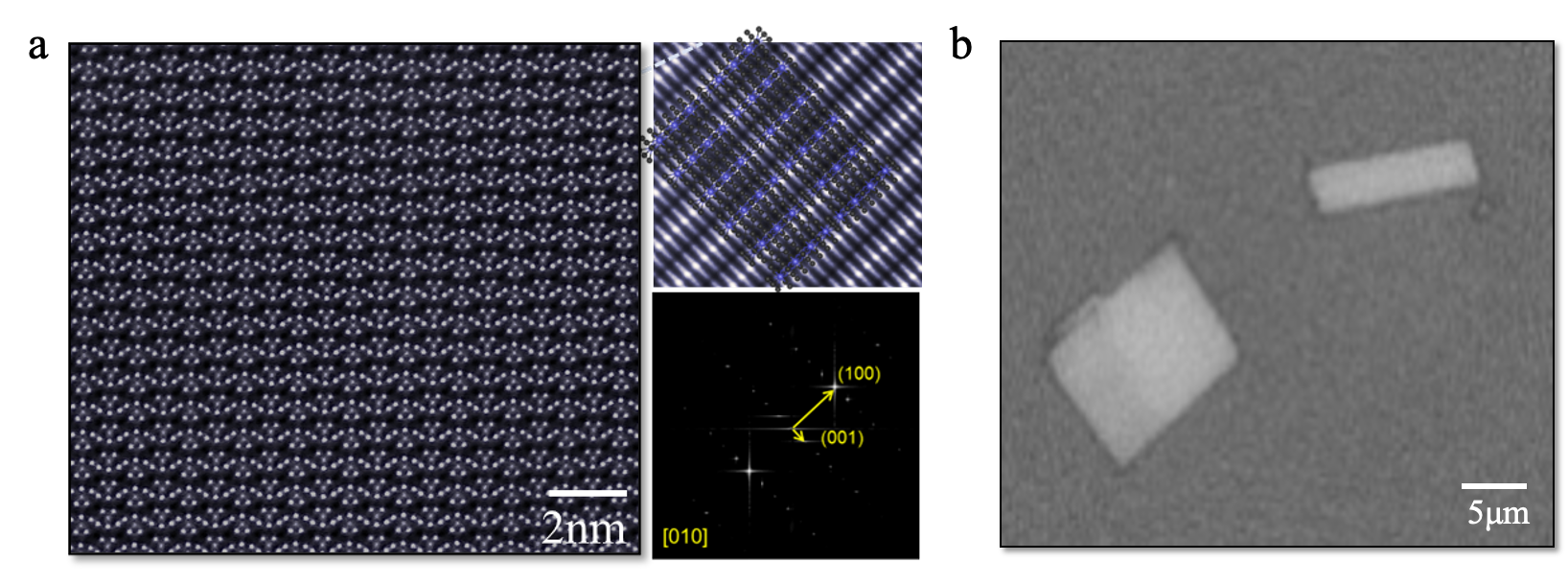


**Supplementary Fig. S1** Low-magnification microscopy image of ZrTe_5_ crystals. This image displays the morphology of the ZrTe_5_ crystals, demonstrating uniform and well-faceted growth. The scale bar represents 5 µm, highlighting the size and shape of the synthesized crystals, which supports the high quality of the material produced through the Chemical Vapor Transport (CVT) method.


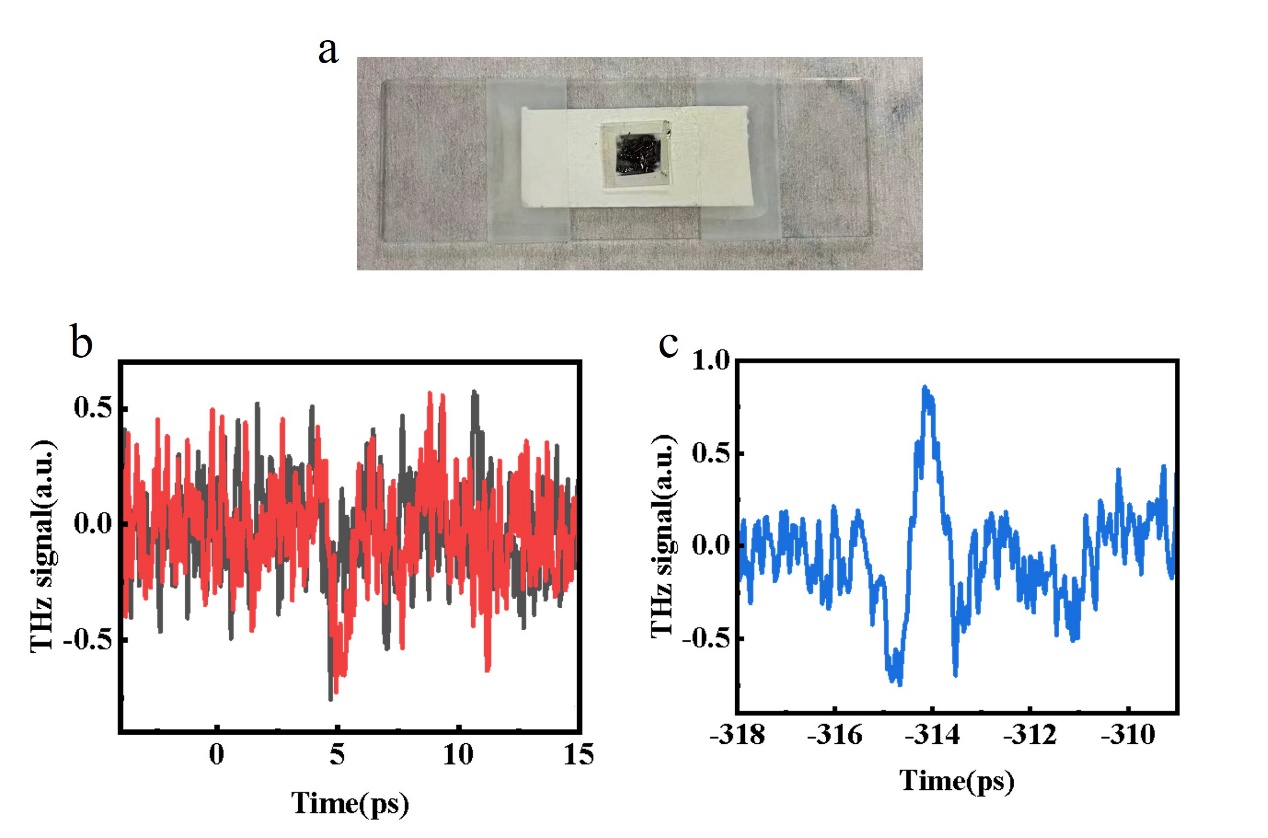


**Supplementary Fig. S2 a** ZrTe_5_ sample mounted on a substrate, prepared for THz signal measurements. This setup is crucial for ensuring accurate and reliable data collection. **b** The time-resolved THz signal, measured in arbitrary units (a.u.) over a timespan of 15 picoseconds (ps). The red and black traces indicate the THz signal’s intensity fluctuations: The signal shows significant noise and rapid fluctuations, suggesting the presence of various dynamic processes within the material. This raw data provides insight into the material’s response to THz radiation, capturing both coherent and incoherent contributions to the overall signal. **c** The zoomed-in view of the THz signal over a shorter time window, from approximately -318 ps to -310 ps. This detailed view highlights a prominent peak around -314 ps, indicating a distinct feature in the THz response.


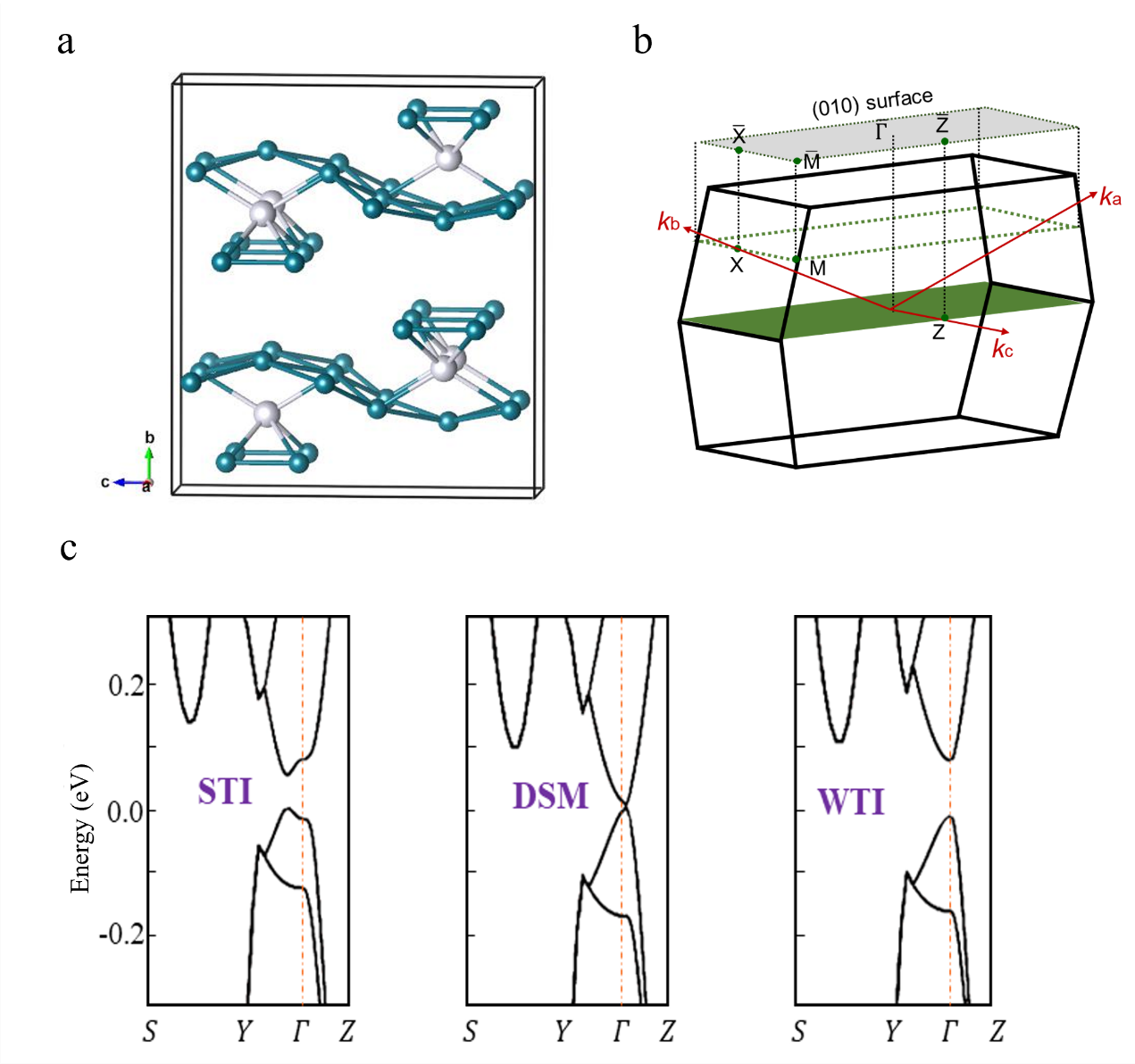


**Supplementary Fig. S3 a** Calculated crystal structure of ZrTe_5_. The calculated crystal structure of ZrTe_5_ is shown, displaying its orthorhombic configuration. The structure consists of layers of Zr and Te atoms arranged in a specific manner, which is crucial for its topological properties. **b** Calculated band structure with the spin-orbit coupling along the high symmetry points of the Brillouin zone(BZ). SOC introduces significant changes in the band dispersion, which are essential for understanding the topological phase transitions in ZrTe_5_. The influence of SOC can lead to the opening of energy gaps and the emergence of topologically protected surface states. **c** The left, middle, and right plots show the energy dispersions before, at, and after phase transition, respectively, accompanied by the band closing. Left Plot (STI - Strong Topological Insulator): Displays the band structure in the strong topological insulator phase, where bands are well-separated. Middle Plot (DSM - Dirac Semimetal): At the phase transition point, the bands touch at the Dirac point, indicating a Dirac semimetal phase with band closing. Right Plot (WTI - Weak Topological Insulator): Shows the band structure after the phase transition, with band inversion and reopening, characteristic of a weak topological insulator.


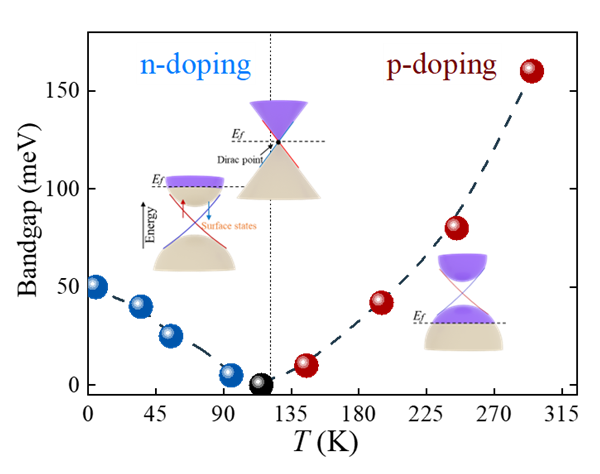


**Supplementary Fig. S4** Bandgap variation (ΔE_g_) of ZrTe_5_ nanosheets as the temperature increases. n-Doping Region (Low Temperatures): At temperatures below the transition point (~135 K), the bandgap decreases, reaching a minimum value. This region is characterized by n-type doping, where electrons are the primary charge carriers. The inset shows the electronic structure near the Dirac point, with surface states present below the Fermi level. The bandgap here is small, indicating a semi-metallic behavior with dominant electron conduction. At the transition temperature (~115 K), the bandgap closes, and the material exhibits a Dirac semimetal phase. This point is critical for the topological phase transition (TPT), where the band structure changes significantly. p-Doping Region (High Temperatures): As the temperature increases beyond the transition point, the bandgap opens up again, and the material enters the p-doping regime, where holes become the primary charge carriers. The inset for the high-temperature region shows an inverted band structure with a reopened bandgap. This behavior is consistent with a topologically non-trivial phase, indicating the presence of topologically protected surface states.


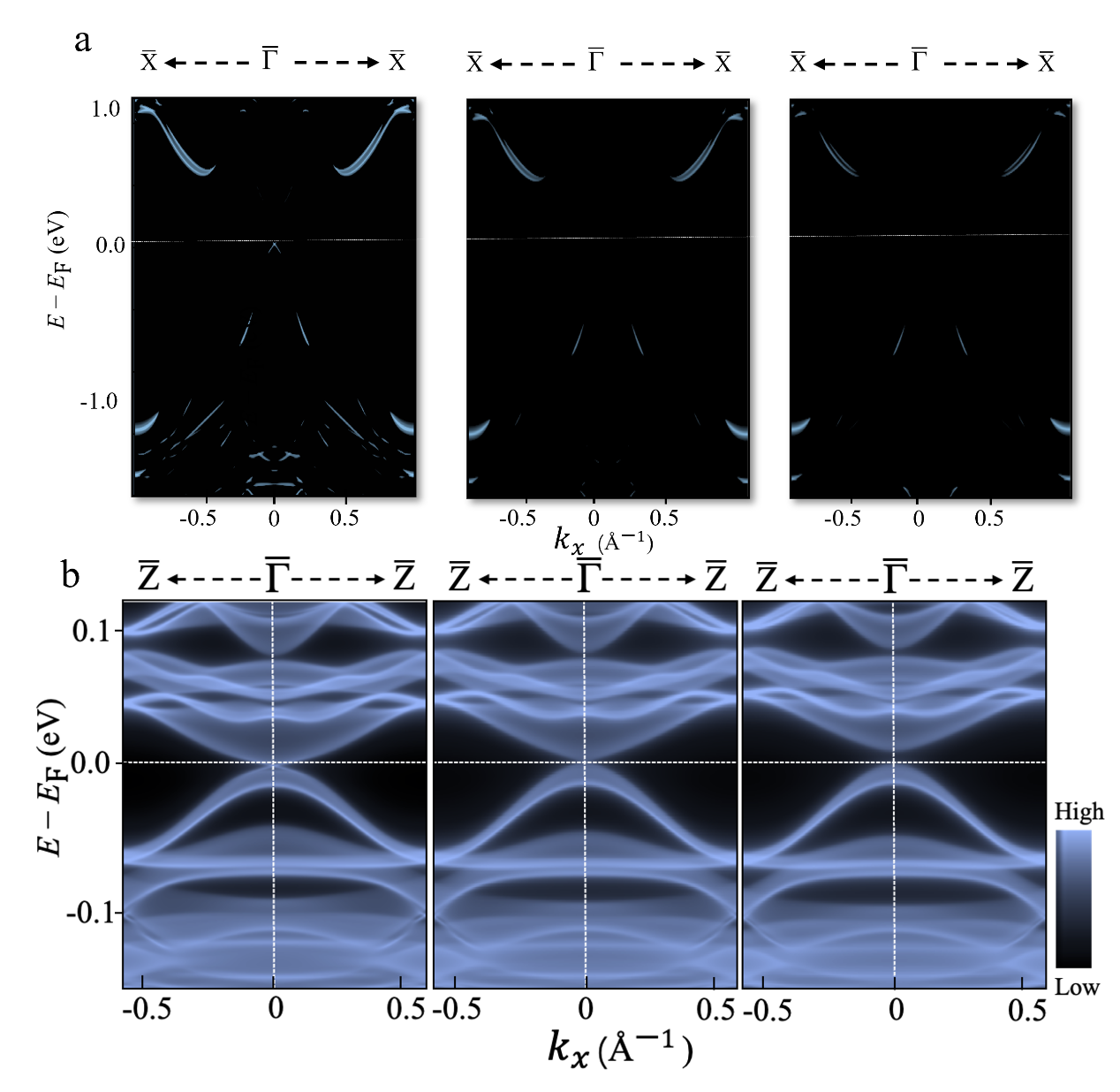


**Supplementary Fig. S5 Progression of topological phase transition with temperature.** **a** The spectral functions on surface states without lattice distortion along the Γ-$\bar{X}$direction. For STI state, all surface state exists on the top surface, and for WTI state, there are surface states on the side surface, while no surface state exists on the top surface. **b** Schematic band diagram of temperature-induced topological phase transition from WTI to DSM to STI states in the k_x_ direction.


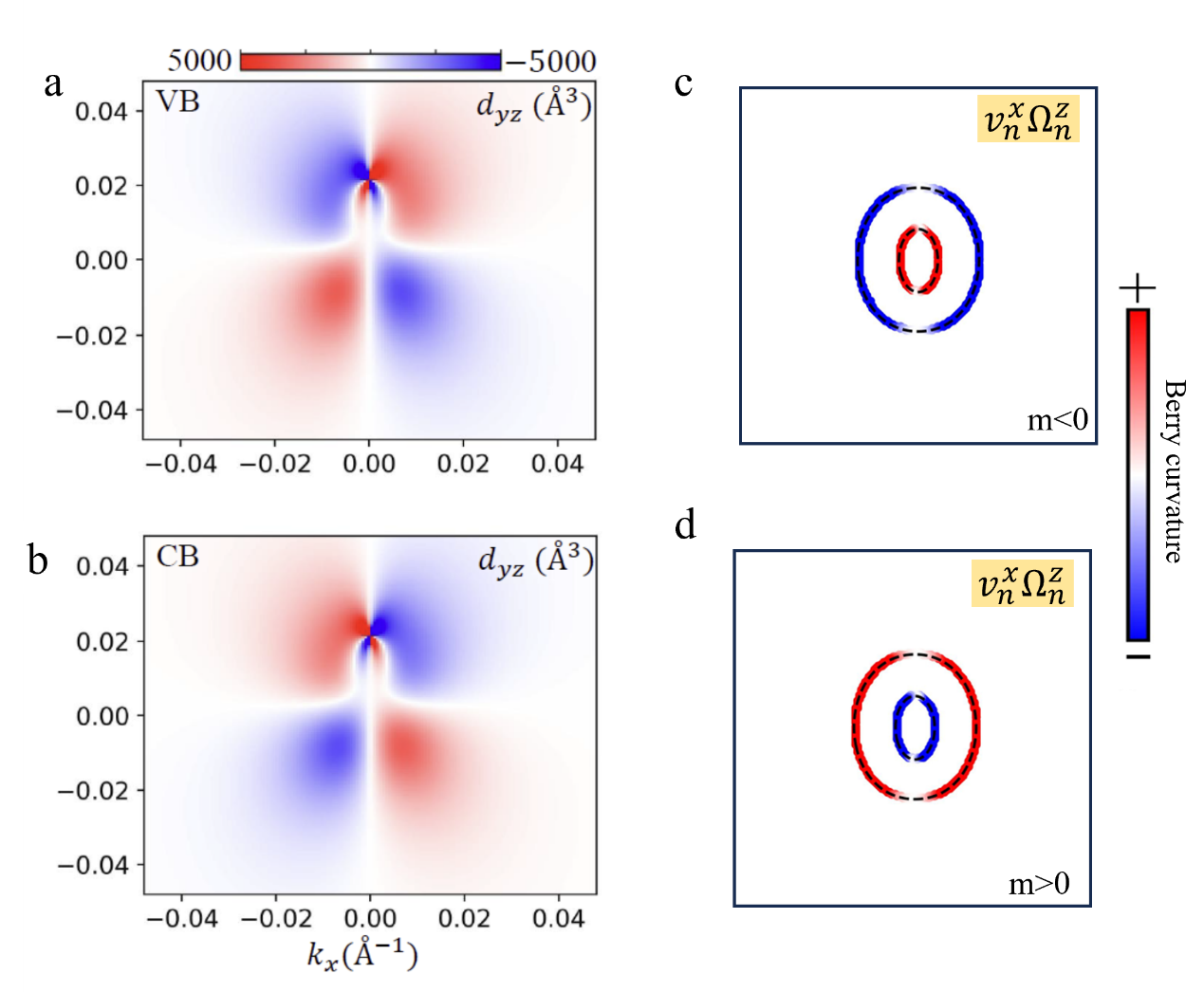


**Supplementary Fig. S6** **a-b** Berry curvature (BC) and BC dipole density of top valence band (VB) and bottom conduction band (CB) projected in k_x_, k_y_ momentum-space while k_z_ = 0. **c-d** illustrate the colormap of the Berry curvature dipole component *D*_xz_ in the *k*_x_-*k*_y_ momentum-space at the Fermi surface for two distinct topological phases, indicated by m<0 and m>0, respectively, without an external bias field. In Fig. S7c, the phase with m<0 exhibits a Berry curvature distribution with a predominately red inner ring and a blue outer ring, while the phase with m>0 in Fig. S7d shows an inverted color scheme, with a blue inner ring and a red outer ring. This inversion between the topological phases explains the polarity change of the DC photocurrent during the topological phase transition (TPT), as *D*_xz_ changes sign with mmm, impacting the direction and magnitude of the photocurrent induced by similar external conditions. This nuanced behavior underscores the sensitivity of topological materials to internal parameters and their profound effect on electronic properties.


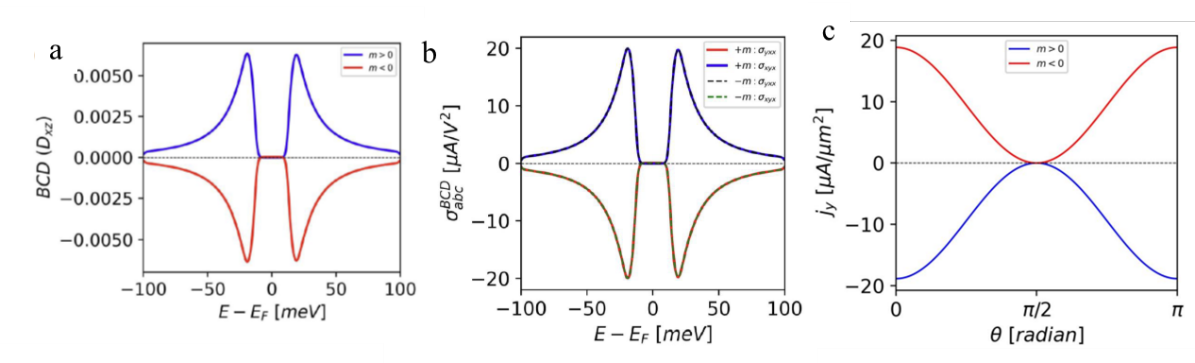


**Supplementary Fig. S7** Berry curvature dipole (BCD) induced photocurrent. **a** The calculated BCD of bulk ZrTe_5_ for positive and negative Dirac mass m. BCD switches sign with changing sign of Dirac mass. The sign change in BCD with Dirac mass inversion indicates that the direction of the induced photocurrent can be controlled by tuning the Dirac mass. This tunability is crucial for designing devices that require precise control over photocurrent direction and magnitude. **b** BCD-induced photoconductivity of ZrTe_5_. Only the in-plane components σ_yxx_ and σ_xyx_ survive for the non-zero BCD component D_xz_. The other in-plane components σ_xyy_ and σ_yxy_ are found to be zero for the vanishing BCD component Dyz. The presence of non-zero σ_yxx_ and σ_xyx_ components suggests that the nonlinear optical response is anisotropic and primarily influenced by the D_xz_ component of the BCD. The absence of contributions from σ_xyy_ and σ_yxy_ underscores the directional dependence of the photoconductivity on the BCD tensor components. **c** The NL photocurrent along y-axis arising from BC dipole, where the electric field polarisation angle θ is with respect to x-axis. The angular dependence of the photocurrent indicates that the polarization of the incident light can be used to modulate the photocurrent’s magnitude and direction.

**
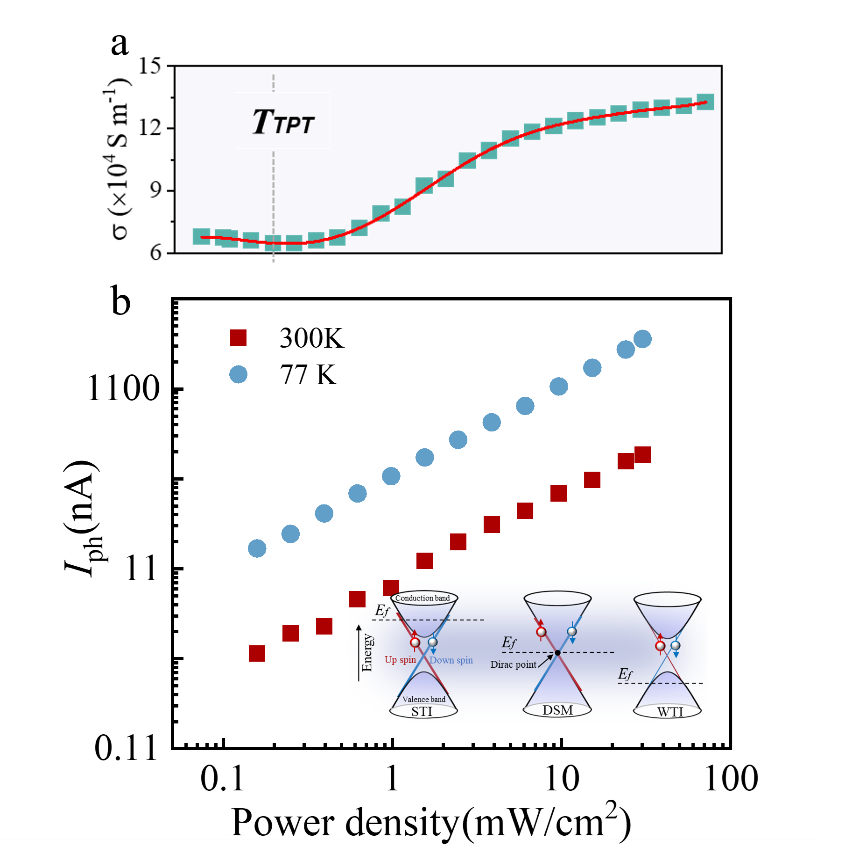
**

**Supplementary Fig. S8** **a** Experimentally measured temperature-dependent conductivity under different conditions, clearly showing the sign change of the photocurrent across the phase transition. The inversion symmetry breaking at T_p_ is due to the staggered displacement of Te atoms along the c axis. **b** Photocurrent I_ph_ vs. Power Density at Different Temperatures. This panel presents the I_ph_ as a function of incident THz power density, comparing results at 77K (blue circles) and 300K (red squares). The inset schematically depicts the band structure of ZrTe_5_, showing transitions from a topological insulator (STI) through a Dirac semimetal (DSM) to a weak topological insulator (WTI). The nonlinear increase in I_ph_ with power density highlights the efficiency of ZrTe5 in generating photocurrent under THz illumination, especially at lower temperatures. The higher I_ph_ at 77K compared to 300K suggests enhanced carrier dynamics and reduced scattering at lower temperatures, leading to more efficient photocurrent generation. The inset’s depiction of band structure transitions underscores the importance of the Dirac semimetal phase in enabling strong BCD and consequently, the observed nonlinear photocurrent.


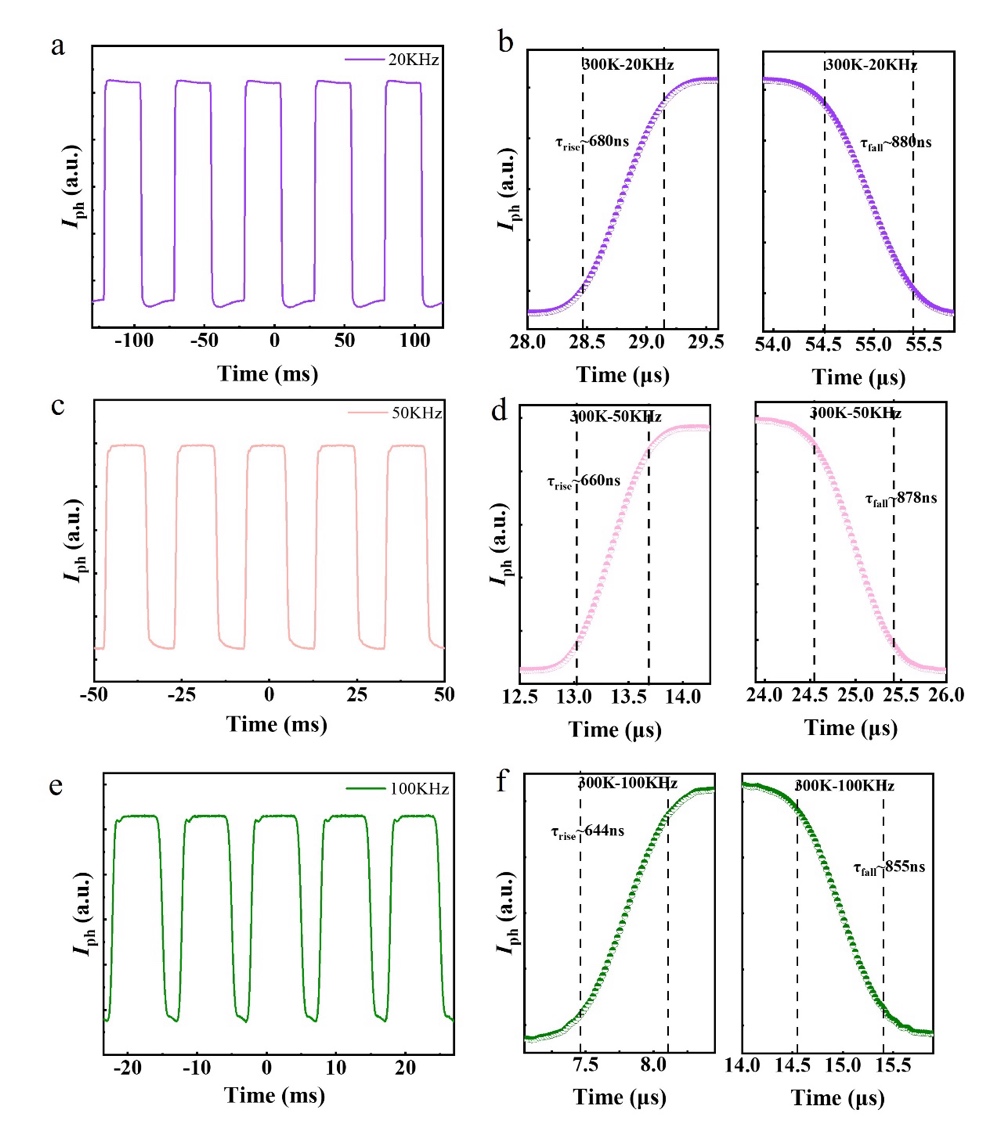


**Supplementary Fig. S9** The response of a ZrTe_5_ THz detector under different modulation frequencies. The data shows response waveforms at 20 kHz (**a-b**), 50 kHz **(c-d)**, and 100 kHz **(e-f)**, along with magnified sections to analyze the response times. This analysis is crucial for understanding the dynamic response and efficiency of ZrTe_5_ as a THz detector.


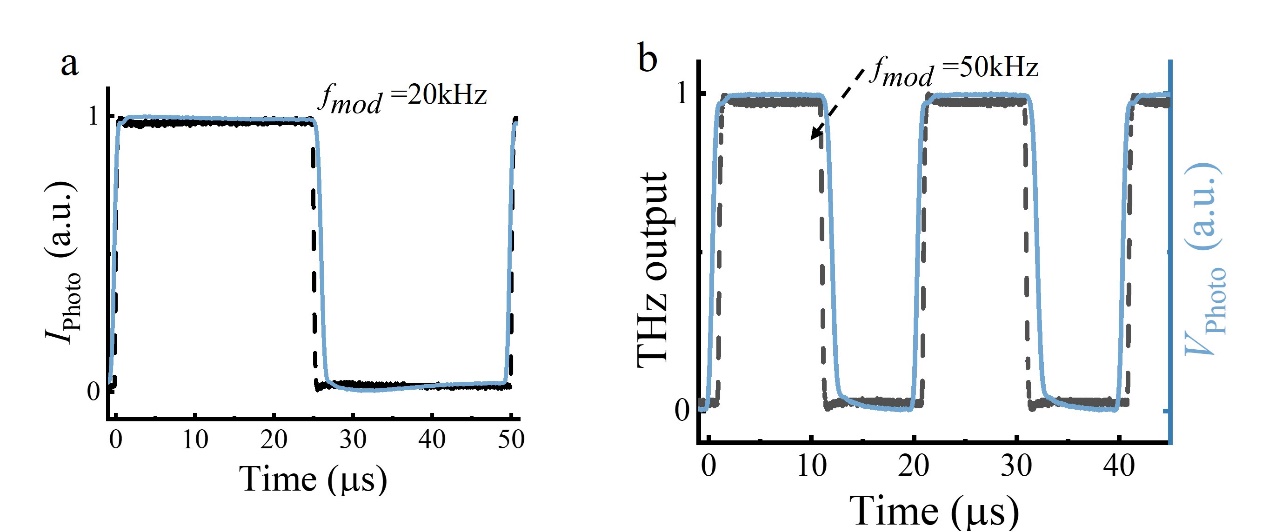
**Supplementary Fig. S10** The response of a ZrTe_5_-based THz detector under different modulation frequencies (20 kHz in **a** and 50 kHz in **b**). The waveform is characterized by a distinct square shape, indicating a sharp rise and fall in photocurrent in response to the modulated THz radiation. This behavior demonstrates the ZrTe_5_’s potential for applications requiring fast and precise THz signal detection. The consistent amplitude of I_ph_ throughout the modulation cycle indicates stability and reliability in the detector’s performance at this frequency. The response at 50 kHz, while still rapid, exhibits a minor lag compared to the lower frequency response. This can be attributed to the inherent response time limits of the material and device structure.


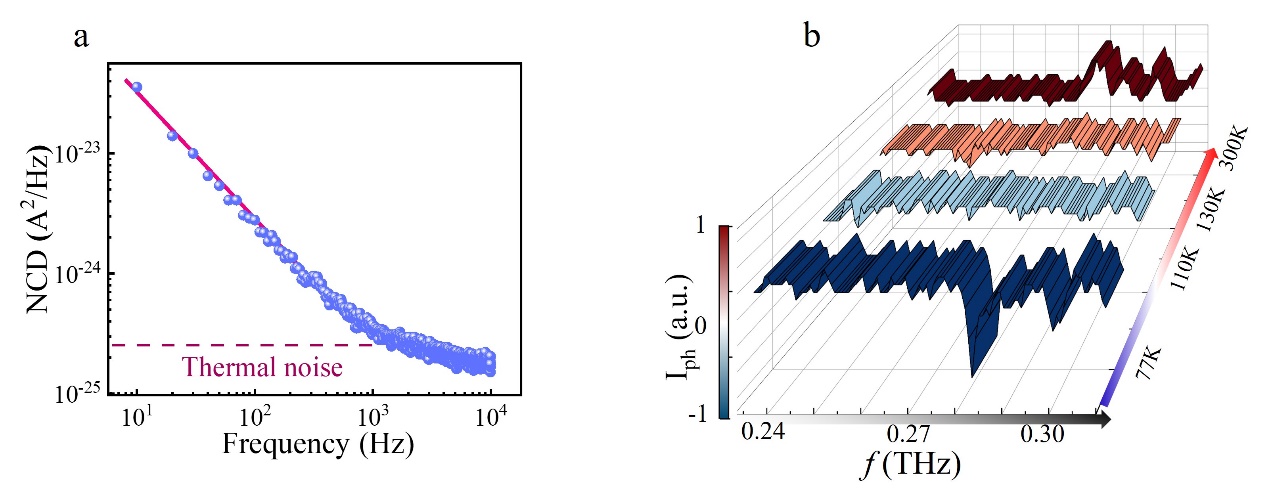


**Supplementary Fig. S11** The device was housed in a temperature-controlled cryostat, shielded from ambient light and electromagnetic interference. The noise current density (NCD) was measured at zero-bias using a Stanford Research Systems (SRS) SR785 Dynamic Signal Analyzer. The device was connected to a low-noise current preamplifier to amplify the signal before it was fed into the analyzer. The input impedance of the preamplifier was set significantly higher than the device resistance to ensure accurate current measurement. The NCD spectrum was recorded over a frequency range of 1 Hz to 20 kHz, and 200 trace averages were taken to obtain a stable noise floor, as shown in Supplementary Fig. S11. The NCD of a ZrTe5-based THz detector as a function of frequency. The solid line represents the measured NCD, while the dashed line indicates the thermal noise floor. The NCD decreases with increasing frequency, following a trend typical of flicker (1/f) noise at low frequencies and transitioning to white noise at higher frequencies. The thermal noise floor represents the fundamental noise limit, below which the detector’s performance cannot improve due to intrinsic thermal fluctuations.


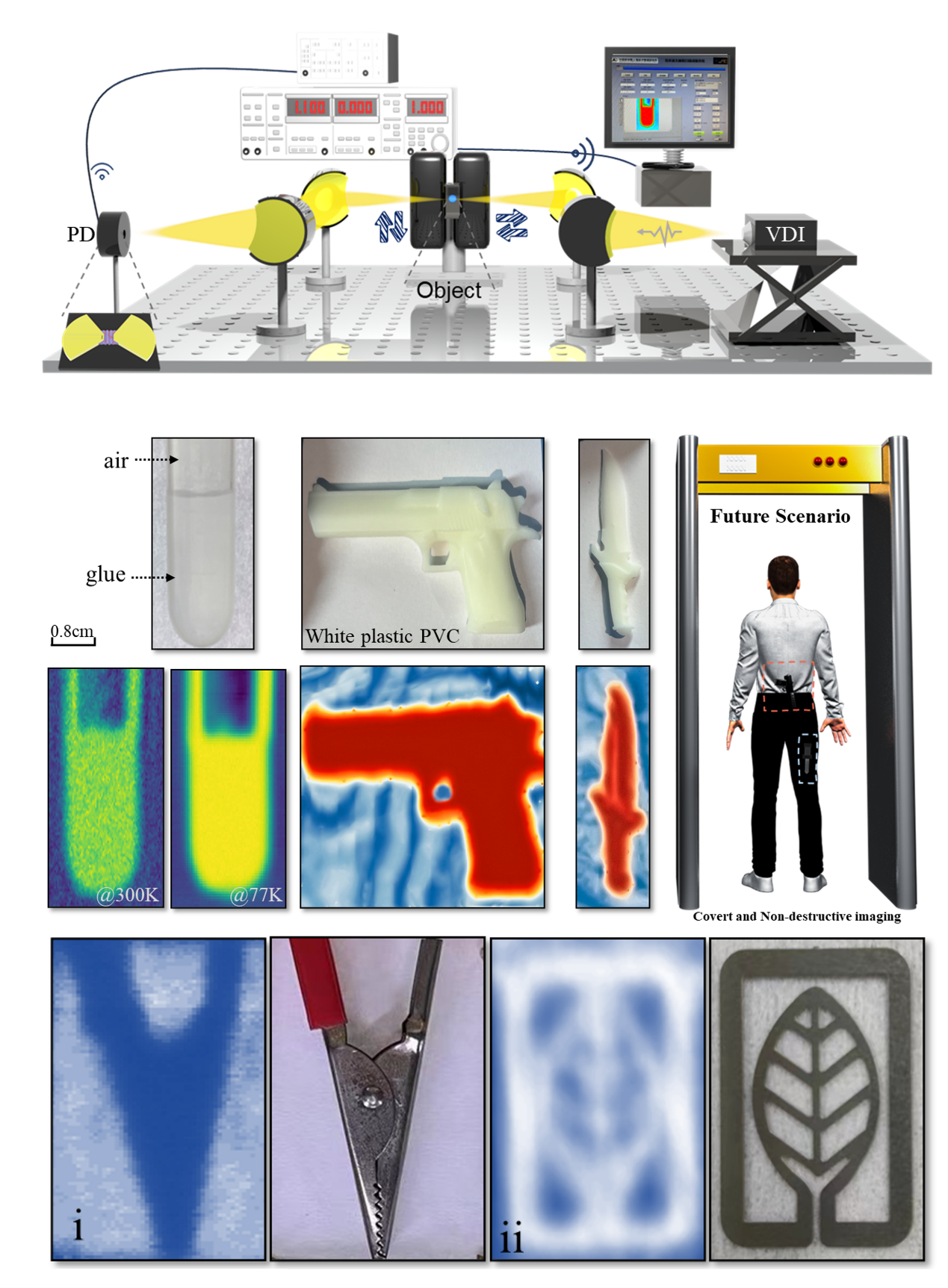


**Supplementary Fig. S12** Non-destructive imaging capabilities in ZrTe_5_-based detector. The ability to detect THz signals non-destructively makes the ZrTe_5_-based detector ideal for applications in quality control, medical imaging, and security scanning. By the scanning step size to 400 μm, the total acquisition time for the 4 cm × 4 cm image was significantly reduced to approximately 50 minutes, while maintaining the diffraction-limited spatial resolution of ~4.9 mm.


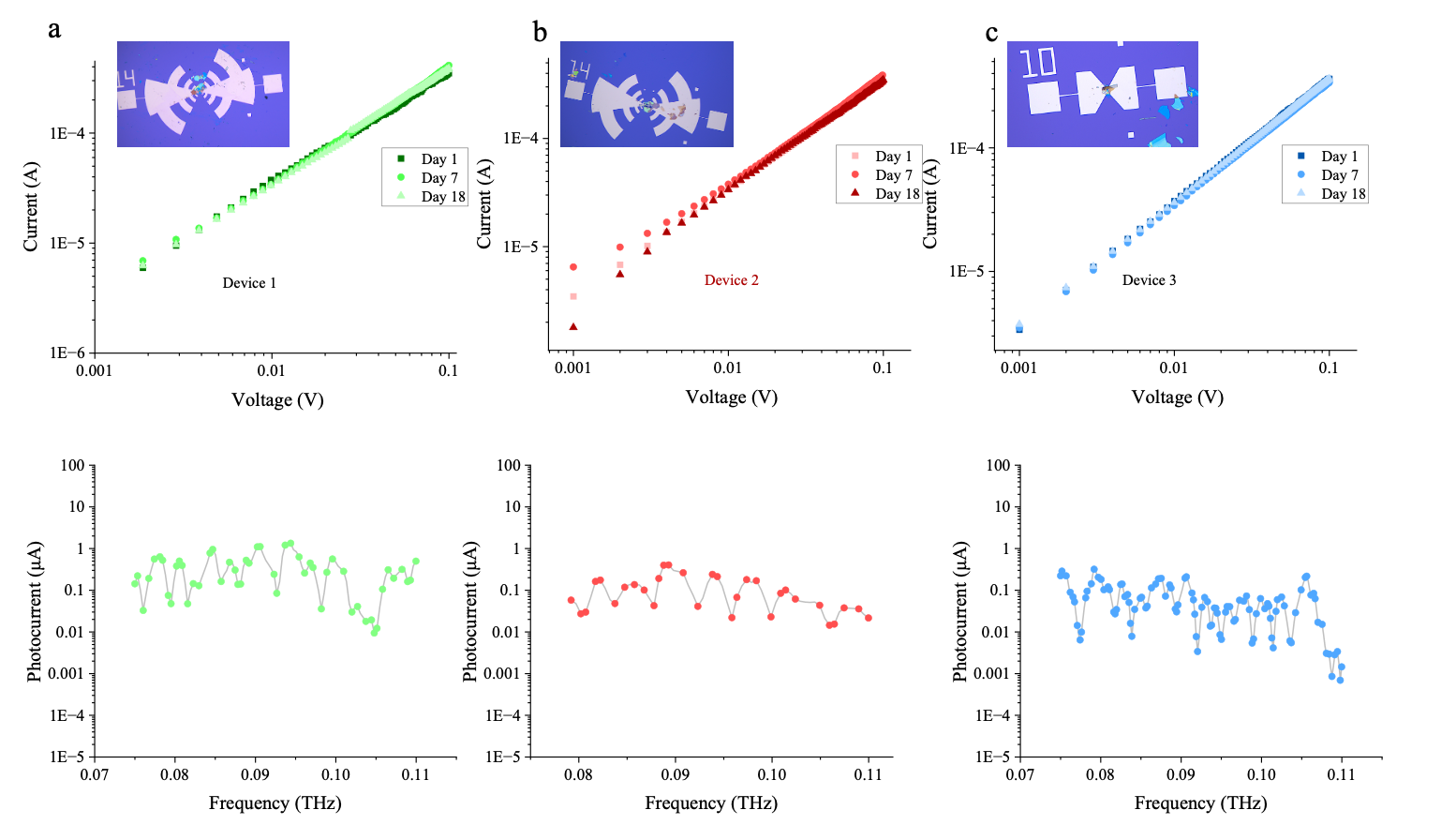


**Supplementary Fig. S13 Device reproducibility and long-term stability.**

a-c Top panels: The plot of the measured current as a function of voltage for three representative devices, respectively. Corresponding long-term stability test. The inset in each panel shows an optical microscope image of the corresponding device. Bottom panels: The terahertz photocurrent was measured periodically at 1 kHz and room temperature. The stable photocurrent values demonstrate the excellent operational stability of the encapsulated devices.

**Reference**

1 Liang, T. et al. Anomalous Hall effect in ZrTe5. Nature Physics **14** (5), 451-455 (2018).

2 Wu, R. et al. Evidence for Topological Edge States in a Large Energy Gap near the Step Edges on the Surface ofZrTe5. Physical Review X **6** (2) (2016).

3 Zhang, Y. et al. Electronic evidence of temperature-induced Lifshitz transition and topological nature in ZrTe5. Nat Commun **8**, 15512 (2017).

4 Zhang, P. et al. Observation and control of the weak topological insulator state in ZrTe5. Nat Commun **12** (1), 406 (2021).

5 Aryal, N., Jin, X., Li, Q., Tsvelik, A. M. & Yin, W. Topological Phase Transition and Phonon-Space Dirac Topology Surfaces in ZrTe_5. Phys Rev Lett **126** (1), 016401 (2021).

6 Zhang, Y., Sun, Y. & Yan, B. Berry curvature dipole in Weyl semimetal materials: An ab initio study. Physical Review B **97** (4) (2018).

7 Facio, J. I. et al. Strongly Enhanced Berry Dipole at Topological Phase Transitions in BiTeI. Phys Rev Lett **121** (24), 246403 (2018).
